# LTR12 promoter activation in a broad range of human tumor cells by HDAC inhibition

**DOI:** 10.18632/oncotarget.9255

**Published:** 2016-05-09

**Authors:** Sonja K. Krönung, Ulrike Beyer, Maria Luisa Chiaramonte, Diletta Dolfini, Roberto Mantovani, Matthias Dobbelstein

**Affiliations:** ^1^ Institute of Molecular Oncology, Göttingen Center of Molecular Biosciences (GZMB), University Medical Center, University of Göttingen, Göttingen, Germany; ^2^ Institute of Human Genetics, Hannover Medical School, Hannover, Germany; ^3^ Dipartimento di Bioscienze, UniversitàdegliStudi di Milano, Via Celoria, Milan, Italy

**Keywords:** factor binding, genome-wide gene expression analysis

## Abstract

A considerable proportion of the human genome consists of transposable elements, including the long terminal repeats (LTRs) of endogenous retroviruses. During evolution, such LTRs were occasionally inserted upstream of protein-coding genes, contributing to their regulation. We previously identified the LTR12 from endogenous retrovirus 9 (ERV9) as a regulator of proapoptotic genes such as *TP63* or *TNFRSF10B*. The promoter activity of LTR12 is largely confined to the testes, silenced in testicular carcinoma, but reactivated in testicular cancer cells by broad-range histone deacetylase (HDAC) inhibitors. Here we show that inhibition of HDAC1-3 is sufficient for LTR12 activation. Importantly, HDAC inhibitors induce LTR12 activity not only in testicular cancer cells, but also in cells derived from many additional tumor species. Finally, we characterize the transcription factor NF-Y as a mediator of LTR12 promoter activity and HDAC inhibitor-induced apoptosis, in the context of widespread genomic binding of NF-Y to specific LTR12 sequences. Thus, HDAC inhibitor-driven LTR12 activation represents a generally applicable means to induce proapoptotic genes in human cancer cells.

## INTRODUCTION

The majority (45 to 69%) of the human genome consists of transposable elements [1, 2]. One class of such elements is represented by long terminal repeat (LTR)-containing retrotransposons, also known as endogenous retroviruses (ERVs), which account for roughly 8% of the human genome [3, 4]. ERVs are considered remnants of ancient retroviral infections that occurred millions of years ago [5, 6]. During a retroviral life cycle, a provirus is inserted into the host's genome [7, 8]. When a provirus is inserted in cells of the germline, it can be passed on in a stable manner according to the Mendelian laws, thus becoming endogenized [7, 9]. As a consequence, full-length ERVs contain the viral genes *gag*, *pol* and sometimes*env*, flanked by two LTRs [6, 7, 10]. However, the vast majority of ERV-derived sequences in the human genome are solitary LTRs [7]. They most probably arose through homologous recombination between two LTRs, resulting in the loss of the retroviral genes between them [11, 12]. LTRs contain important gene regulatorysequence elements, such as promoters and enhancers [6, 13]. When inserted in close proximity to a host gene, LTRs can influence its expression pattern [14]. However, little is known about the factors that regulate the promoter activity of LTRs, and about the functional consequences of such LTR activities in human cells.

Since the mobility of transposable elements is potentially harmful to the host through disruption or de-regulation of genes, various mechanisms evolved in order to restrict their general activity. Among these are DNA methylation, small inhibitory RNAs and histone modifications [15–18]. Such modifications can diminish the promoter activity of retroviral LTRs, interfering with the transcription of downstream virus genes and thus preventing the spread of ERVs through the genome. However, concepts for the specific and dynamic regulation of single LTRs in human cells are lacking.

An LTR of the HERV-9 (ERV9) family was previously characterized in our laboratory as to its promoter activity, in particular at two genomic locations [19, 20]. This LTR is termed LTR12 according to the database of repetitive elements Repbase [21]. First, we found that LTR12 confers the tissue-specific expression of GTAp63, a testicular isoform of the p53 tumor suppressor homologue p63 derived from the gene *TP63*, in male germ cells [20]. Furthermore, an LTR12 inserted upstream of *TNFRSF10B* drives its expression in testis [19]. TNFRSF10B, also known as Killer/DR5, encodes a death receptor that transmits the proapoptotic signal of Trail [22, 23].

Interestingly, transcription of LTR12-driven *TNFRSF10B* and *TP63*was enhanced by more than 1000-fold in testicular cancer cells upon treatment with the HDAC inhibitors (HDACi) Trichostatin A (TSA) as well as the structurally similar suberoylanilidehydroxamic acid (SAHA) [19, 20]. Functionally, this enhanced LTR12 promoter activity resulted in elevated levels of the *TNFRSF10B* gene product, death receptor 5 (DR5), and testicular cancer cell death.

The trimeric nuclear factor Y (NF-Y) associates with LTR12. NF-Y is composed of the three subunits named NF-YA, NF-YB and NF-YC. While NF-YA confers sequence-specificity for the DNA motif CCAAT, NF-YB and NF-YC have histone-like structural features to bind DNA with broader specificity [24, 25]. Binding of NF-Y to an LTR12 upstream of the beta-globin locus control region can influence its enhancer activity through the recruitment of additional transcription factors [26]. Moreover, a genome-wide search for NF-Y binding sites in the human genome revealed clustering at HERV LTRs, in particular at LTR12s and MLT1 LTRs [27]. The functional relevance of this association, however, remains to be determined. In general, NF-Y is implied in gene activation as well as gene repression, and it can associate with histone acetyl transferases (HATs) but also deacetylases [28–32]. While acetylation of histones by HATs is believed to mostly relax the chromatin and render it more accessible to the transcription machinery, deacetylation opposes these effects [33, 34]. Histone deacetylases, which remove acetyl groups from histones, also have non-histone substrates such as transcription factors and chaperone proteins [35, 36]. The molecular responses to alterations in HDAC activity range from apoptosis, migration and differentiation to angiogenesis [37–39]. HDACs are commonly divided into four classes. HDACs of class I (HDAC1, 2, 3 and 8), IIa (HDAC4, 5, 7 and 9), IIb (HDAC10 and 6) and IV (HDAC11) each carry a zinc ion(Zn^2+^) at their active sites [40–42]. Hence, these HDACs are characterized as zinc-dependent HDACs and can be inhibited by agents that compete with the substrate for interaction with the zinc ion [42]. In some cancers, particular HDACs are overexpressed [43], and inhibition of HDAC activity can represent an efficient anticancer treatment. Aside from the HDAC inhibitors SAHA and romidepsin, which are approved for the treatment of cutaneous T-cell lymphoma [42, 44, 45], various other HDAC inhibitors are currently undergoing clinical trials for the treatment of different tumors, including lymphomas and solid tumors [42, 44]. Some of these HDAC inhibitors are specific for certain classes or even individual HDACs [37, 44].

Here, we sought to determine how HDAC inhibitors increase LTR12 promoter activity and whether these mechanisms are also accessible in cancer cells that were not derived from testicular carcinoma. We observed strong enhancement of LTR12-driven gene transcription upon treatment with HDAC inhibitors that selectively target HDAC 1/2/3. The transcription factor NF-Y was found to be involved in LTR12 regulation. Of particular note, LTR12 promoter activity was not only observed in testicular cancer cells but was strongly increased by HDAC inhibitors in a broad variety of human cancer cells.

## RESULTS

### Inhibitors of HDACs 1-3 induce LTR12 promoter activity

**Figure 1:**
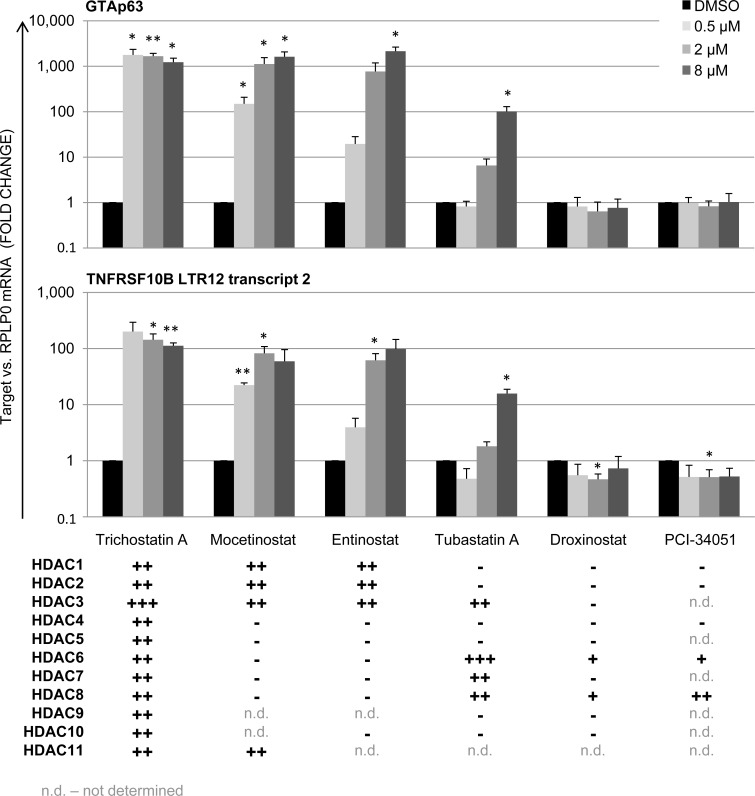
LTR12 induction by inhibitors of HDACs 1-3 in GH cells treated with HDAC inhibitors from different chemical classes Cells were treated with increasing concentrations of each inhibitor (0.5 μM, 2 μM, 8 μM) for 18 h. Subsequently, relative gene expression was assessed by qRT-PCR. The levels of mRNA corresponding to the *TP63* isoform GTAp63 and to the LTR transcript 2 of *TNFRSF10B* are depicted. A significant increase in transcription of the LTR12-driven isoforms of *TP63* and *TNFRSF10B* was observed upon treatment with Trichostatin A, Entinostat, Mocetinostat and Tubastatin A. For all tested HDAC inhibitors, their specificities for each known HDAC are indicated, according to the average IC50 values as indicated at PubChem (https://pubchem.ncbi.nlm.nih.gov/). mRNA levels were normalized to *RPLP0* and are shown as a fold change of DMSO-treated control cells. Error bars represent the standard deviation, SD (*n* = 3). *, *p* < 0.05; **, *p* < 0.01; ***, *p* < 0.001. IC50 values for the HDAC inhibitors are represented as follows: +++ < 10 nM; ++ 10 nM-1 μM; + 1-10 μM; − > 10 μM. n. d., not determined.

Based on our previous observations that LTR12-driven gene transcription is strongly induced bythe hydroxamate HDAC inhibitors TSA and SAHA [19, 20], we now tested a panel of HDAC inhibitors from different chemical classes as to their influence on LTR12 promoter activity. Testicular cancer-derived GH cells were treated with 0.5/2/8 μM of HDAC inhibitors for 18 h. Subsequently, the relative gene expression levels of LTR12-driven *TP63* (GTAp63) and *TNFRSF10B* were assessed by qRT-PCR. Aside from TSA, LTR12 promoter activity was significantly increased upon treatment with Entinostat, Mocetinostat and to a lesser extent withTubastatin A (Figure 1). While TSA and SAHA bind all eleven zinc-dependent HDACs [46], Mocetinostat and Entinostat are selective inhibitors for HDACs 1, 2 and 3 [37]. Thus, inhibition of HDACs 1-3 appears to make the largest contribution to the activation of LTR12. Moreover, we conclude that benzamide HDAC inhibitors, as well ashydroxamic acid HDAC inhibitorseach activate LTR12. This largely precludes off target effects but confirms the notion that HDAC inhibition is indeed the key mechanism of LTR12 activation.

### HDAC inhibitors induce LTR12 promoter activity in cells from various tumor species

**Figure 2:**
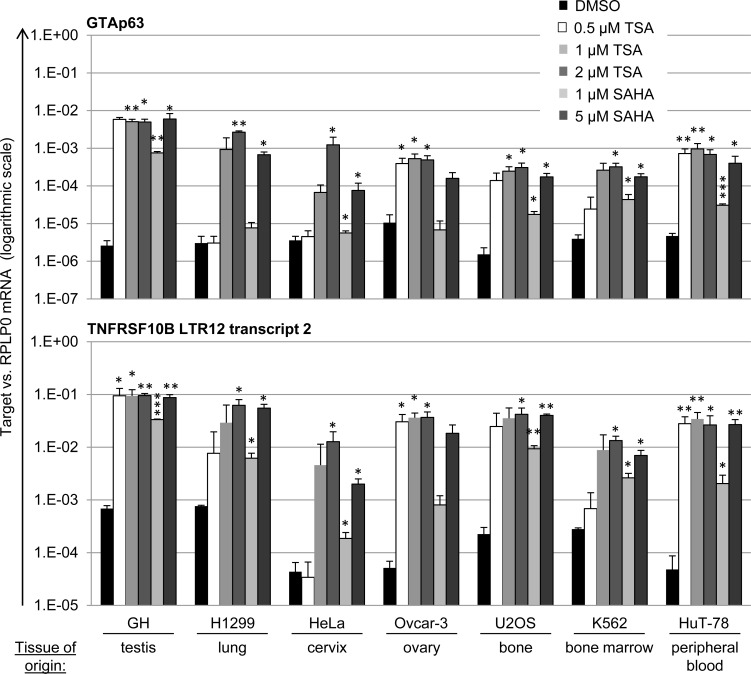
LTR12 induction by HDAC inhibitors in cell lines from various tumor species Human cell lines derived from varioustumor origins were treated with the HDAC inhibitors Trichostatin A (TSA) or suberoylanilidehydroxamic acid (SAHA) at increasing concentrations (0.5 μM,1 μM, 2 μM for TSA and 1 μM, 5 μM for SAHA) for 18 h, followed by qRT-PCR analysis. Cells treated with DMSO alone were used as controls. mRNA levels were normalized to RPLP0. The mRNA levels corresponding to LTR-driven GTAp63 and TNFRSF10BLTR transcript 2 [19] are depicted. Strong increases in transcription for both LTR-driven transcripts were observed in all tested cell lines. For a comparison with overall levels of TP63 and TNFRSF10B mRNA, see Supplemental Figure S1. The cell lines were GH (testicular cancer), H1299 (lung carcinoma), K562 (leukemia), U2OS (osteosarcoma), HeLa (cervical carcinoma), Ovcar-3 (ovarian carcinoma) and HuT-78 (cutaneous T-cell lymphoma). Error bars represent the SD (*n* = 3). *, *p* < 0.05; **, *p* < 0.01, ***; *p* < 0.001.

LTR12-driven transcription is present in normal testis, but silenced in testicular cancer cells [20]. However, treatment with HDAC inhibitors results in strong de-repression of the LTR12 promoter activity [19, 20]. We now asked whether this mechanism is also accessible in tumor cells derived from tissues other than testis. A panel of human cancer cell lines was treated with TSA (0.5/1/2 μM) and SAHA (1/5 μM) over a period of 18 h. This included GH (testis cancer), H1299 (lung carcinoma), HeLa (cervical carcinoma), Ovcar-3 (ovarian carcinoma), U2OS (osteosarcoma), K562 (leukemia) and HuT-78 (cutaneous T-cell lymphoma) cells. Of note, cutaneous T-cell lymphoma is the tumor entity where SAHA (alias Vorinostat) represents the FDA-approved treatment option [47]. Assessment of LTR12-driven gene transcription revealed a strong (up to several hundred-fold) increase in the transcription of both GTAp63 (Figure 2, upper panel) and LTR12-driven *TNFRSF10B* (Figure 2, lower panel) in all cell lines. In parallel, the total levels of *TAp63* and *TNFRSF10B* were quantified, including transcripts starting from the previously described sites within non-LTR, “host” gene promoters. Here, the increase in transcription was still observed, but less strongly (Suppl. Figure S1). We propose that the LTR12 promoters, but not the additional promoters of the genes under study, are activated by HDAC inhibitors, resulting in a moderate increase when analyzing the mixed population of mRNAs. In conclusion, HDAC inhibitors strongly activate LTR12-driven transcription not only in testicular cancer cells, but in a variety of cell lines derived from multiple tumor species.

### HDAC inhibitors induce the promoter activities of LTR12 but not of LTRs from different endogenous retroviruses

**Figure 3:**
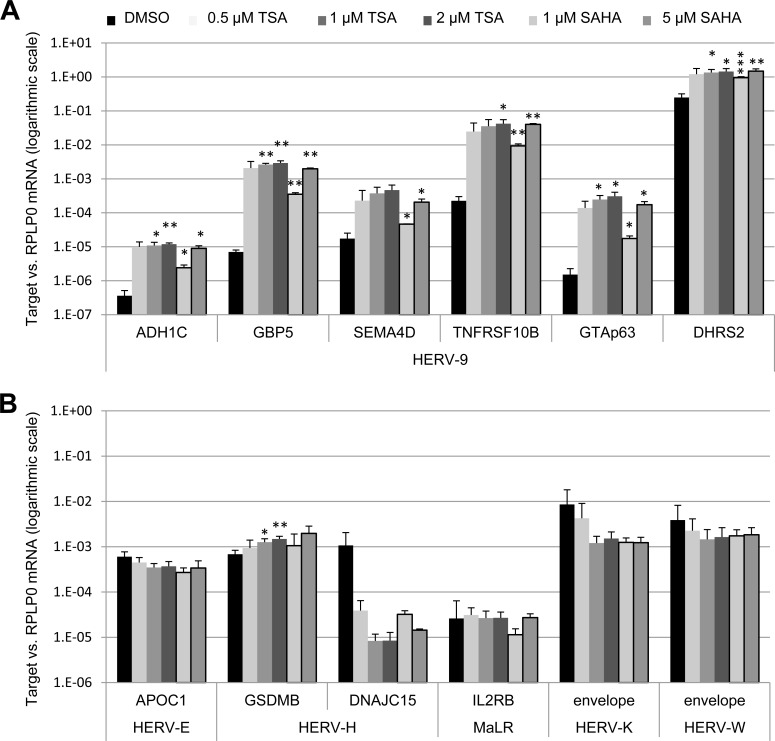
HDAC inhibitors inducing exclusively LTR12 but not other LTRs from endogenous retroviruses U2OS cells were treated with the HDAC inhibitors Trichostatin A (TSA) or suberoylanilidehydroxamic acid (SAHA) or the DMSO solvent alone, using increasing concentrations (0.5 μM, 1 μM, 2 μM for TSA and 1 μM, 5 μM for SAHA) for 18 h, followed by qRT-PCR with normalization to *RPLP0*. The mRNA levels corresponding to LTR12-driven isoforms of different cellular genes [A] and to cellular genes driven by LTRs from other ERV families and envelope genes of the endogenous retroviruses HERV-K and HERV-W [B] are depicted. Strongly enhanced transcription was observed for all six genes driven by an LTR12 (HERV-9 LTR). In contrast, little or no enhancement by TSA was found for the transcription of genes driven by LTRs other than LTR12, as we found previously for testicular GH cells [19]. Error bars represent the SD (*n* = 3). *, *p* < 0.05; **, *p* < 0.01; ***, *p* < 0.001.

Next, we addressed the question whether additional LTRs from various endogenous retroviruses might be subject to activation by HDAC inhibitors. We treated U2OS cells with TSA (0.5/1/2 μM) and SAHA (1/5 μM) and assessed the transcription of six LTR12-driven host genes but also HERV-E-driven *APOC1*, two HERV-H-driven host genes, MaLR-driven *IL2RB*, and viral envelope genes of HERV-K and HERV-W. As a result, we observed a specific induction of LTR12-regulated transcription (Figure 3A) [19] but not that of other LTRs in response to HDAC inhibition (Figure 3B), as we had seen previously with testicular GH cells [19]. Hence, the strong enhancement of LTR promoter activity by HDAC inhibitors is specific for ERV9 LTRs but does not pertain to different human ERV promoter elements. On the other hand, LTR12s do not depend on their specific integration site to function as HDAC inhibitor-responsive promoters.

### Various LTR12s in the human genome show consensus NF-Y binding sites and associate with NF-Y

**Figure 4:**
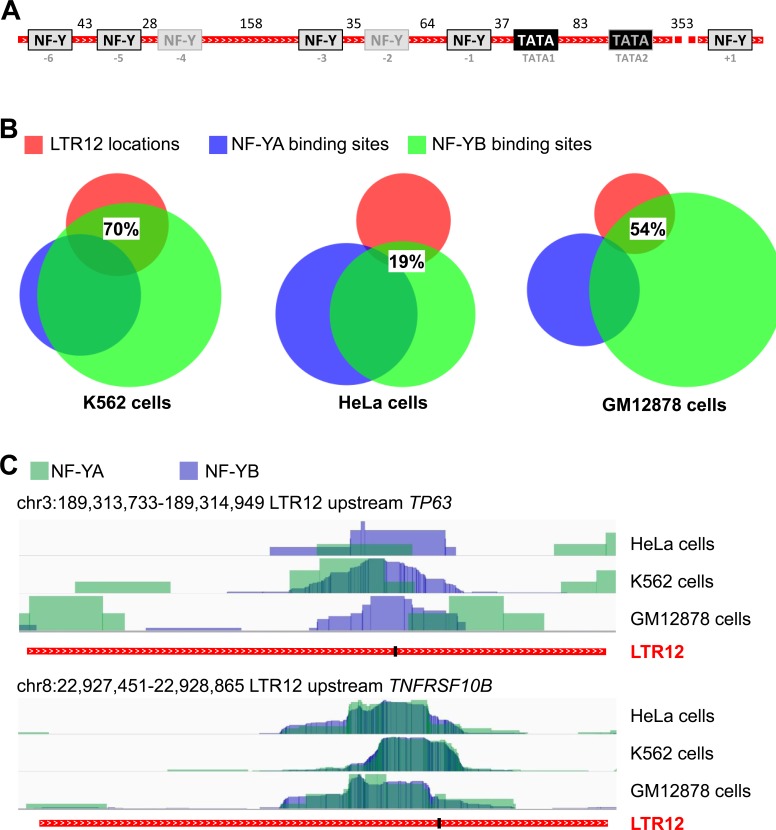
NF-Y binding sites within LTR12 **A.** Predicted NF-Y binding sites within LTR12. NF-Y binding sites (grey boxes) within HDAC inhibitor-responsive LTR12 promoter elements (red), as predicted by *in-silico* analysisusing the ALGGEN PROMO tool (http://alggen.lsi.upc.es/) [61, 62]), are indicated. Moreover, TATA boxes (AATAAA) are shown. The numbers represent the average distances between binding sites in nucleotides upon alignment of the LTR12s using CLUSTALW, cf. Supplemental Table S1. Twenty-two LTR12s were included in the analysis. The alignment is shown in Supplemental Figure S2. Boxes framed in black indicate binding sites that are present in more than 65% of all analyzed LTR12s, whereas grey frames indicate less frequent binding sites. **B.** Confirmed NF-Y binding sites are enriched within LTR12s. ChIP-seq data that indicate binding of the NF-Y subunits alpha (NF-YA) and beta (NF-YB) [27] were retrieved from GEO and analyzed for binding sites with LTR12 locations. NF-Y binding was analyzed in K562 cells, HeLa-S3 cells, and GM12878 cells, as detailed in Supplemental Table S3. Note that not all locations are detectably bound by both subunits. The association of NF-Y to LTR12s differs between the three cell lines. Between 19% and 70% of the analyzed LTR12s were bound by NF-Y. In contrast, LTRs from different ERVs have far lower occupation with NF-Y, as depicted in Supplemental Figure S3. **C.** ChIP-Seq tracks of the LTR12s upstream of *TP63* and *TNFRSF10B.* ChIP-seq data for binding of the NF-Y subunits alpha (NF-YA, green) and beta (NF-YB, blue), according to [27], were retrieved from GEO for HeLa, K562 and GM12878 cells. Bar charts of binding intensities at the LTR12 promoters upstream of *TP63* (upper panel) and *TNFRSF10B* (lower panel) are presented. The location of the TATA1 box (see Figure 4A) within each LTR12 is indicated by a black bar.

Based on the uniform activation of the ERV9-LTR12 but no other LTRs by HDAC inhibitors, we considered the possibility that a common LTR12-binding transcription factor might be involved in its promoter activation. One transcription factor that was previously implied in LTR12 promoter regulation is nuclear factor Y (NF-Y) [26, 48]. *In-silico* prediction of putative NF-Y binding sites within twenty-two different HDAC inhibitor-responsive LTR12s (Suppl. Figure S2) revealed the presence of seven sites on average within each LTR12. Alignment of the analyzed LTR12 sequences showed that the position of some NF-Y binding sites was conserved throughout most sequences (Figure 4A; Suppl. Figure S2). NF-Y binding site “-1”, for example, was predicted in 91% of all analyzed sequences and in a constant distance of 37 nucleotides from the first TATA box (Suppl. Table S2). In line with our prediction, a recent genome-wide Chromatin Immunoprecipitation (ChIP) study of NF-Y binding sites in three human cell lines revealed that a bulk of these sites overlapped with endogenous retroviral LTRs [27]. To identify the occupancy of LTR12s with NF-Y, we retrieved the NF-Y binding sites identified by Fleming *et al.* [27] and searched them for regions corresponding to LTR12 sequences. In HeLa-S3 cells, about 19% of all LTR12 locations in the human genome were found to be bound by an NF-Y subunit. Even more impressively, 70% of LTR12s bound NF-Y in K562 cells (Figure 4B). For GM12878 cells, 54% of all LTR12 sites were occupied by NF-Y. This included the LTR12s upstream of *TP63* and *TNFRSF10B*, as depicted in Figure 4C. We also searched for NF-Y-associated sites with LTRs from other HERV families, e. g. LTR2 of the HERV-E family, and observed a much lower occupancy with NF-Y (between 0% and 0.5%; Suppl. Figure S3). Taken together, we conclude that NF-Y is not only predicted to bind LTR12s, but actually bound to LTR12 promoter elements in different human cell species with high abundance and specificity.

### NF-Y binds LTR12, and the interaction is fortified by HDAC inhibition

**Figure 5:**
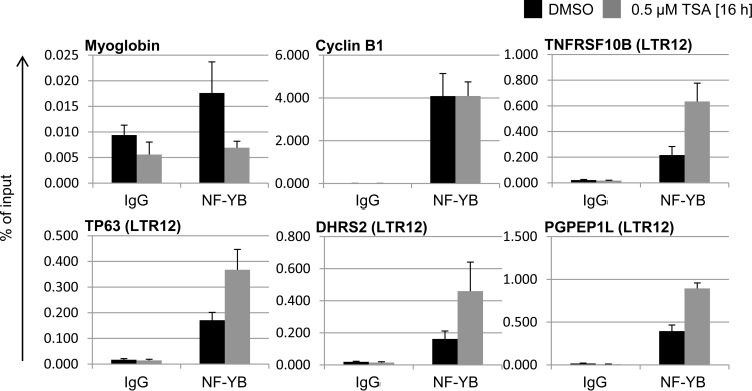
Increased binding of NF-YB to LTR12s after treatment with TSA GH cells were treated with 0.5 μM TSA or the DMSO solvent as indicated. Subsequently, Chromatin immunoprecipitation (ChIP) was performed with antibodies to NF-YB or a pre-immune control antibody. The DNA corresponding to the indicated promoters was quantified by real-time PCR and displayed in relation to the amount of input DNA. Bars indicate the standard error from three independent experiments.

To determine whether NF-Y was also present on LTR12 in a testicular context, and whether its binding pattern was subject to regulation by HDAC inhibitors, we assessed NF-Y chromatin binding in testicular cancer cells. Coordinates of NF-Y peaks within LTR12 sequences were first retrieved from the genome-wide analyses in HeLa, GM12878 and K562 cells (Suppl. Table S3). Of note, many but not all LTR12 sites were occupied by NF-Y; the selectivity of binding might be determined by other transcription factors and/or the chromatin structure. According to the sites of maximum NF-Y association, primers for amplification of the LTR12s adjacent to *DHRS2*, *PGPEP1L*, *TNFRSF10B* and *TP63* were designed, which gave rise to the expected PCR products when tested on genomic DNA. After treatment with TSA or its solvent DMSO, testicular cancer cells were subjected to chromatin harvesting and immunoprecipitation with an antibody recognizing NF-Y subunit beta (NF-YB). The inactive promoter region of the *myoglobin* gene MB was analyzed as a negative control. CCNB1/Cyclin B1, which is strongly bound by NF-Y [49], was detected as a positive control. In untreated cells, NF-YB was bound to CCNB1, as well as to all four analyzed LTR12 promoter elements (Figure 5). Upon treatment with TSA, the interaction of NF-Y with all four LTR12s increased between two- and four-fold. This indicates that NF-Y is not only present at the endogenous retroviral promoter elements in testicular cancer cells, but that its binding increases in response to treatment with TSA.

### NF-Y is a determinant of LTR12 promoter activity and TSA-mediated apoptosis

**Figure 6:**
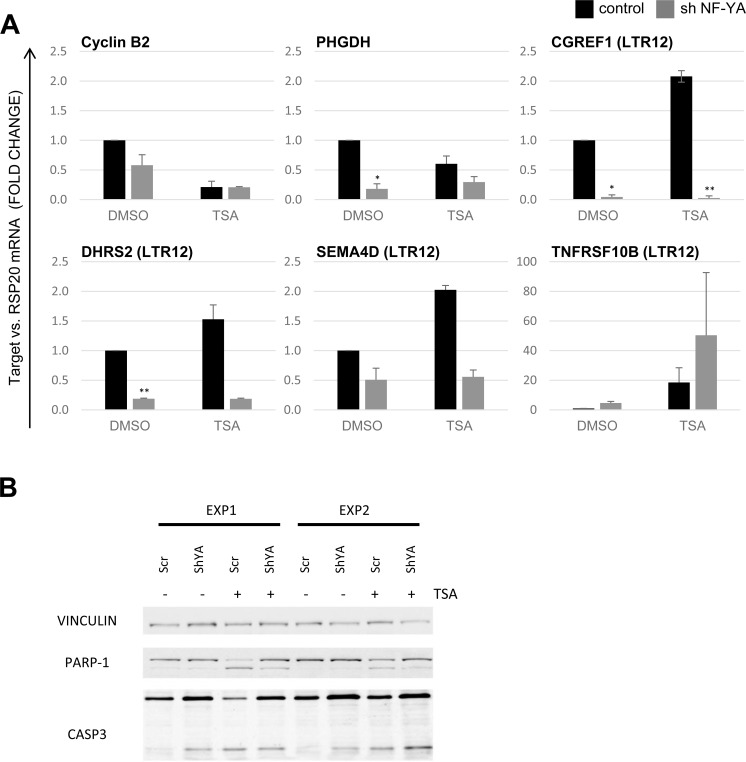
Positive regulation of LTR12 activity by NF-Y **A.** HeLa cells were depleted of NF-YA by shRNA as described [49, 64], and depletion was confirmed as shown in cf. Supplemental Figure S4A. The cells were then treated with 2 μM TSA for 18 h, and relative gene expression was assessed by qRT-PCR with normalization to *RSP20*. The transcription levels of Cyclin B2/CCNB2, *PHGDH* (positive controls responding to NF-Y depletion) as well as four LTR12-driven genes *(CGREF1, DHRS2, SEMA4D, TNFRSF10B)* are depicted. A significant decrease in transcription of the LTR-driven isoforms was observed upon removal of NF-YA for *CGREF1* and *DHRS2* as well as for *PHGDH*.SD (*n* = 2). *, *p* < 0.05; **, *p* < 0.01; ***, *p* < 0.001. **B.** Upon treatment as in A, caspase cleavage was determined by immunoblot analysis. Vinculin staining served as a loading control. Poly-ADP Ribose Polymerase (PARP) and Caspase 3 were detected at two different molecular weights each. The upper bands represent the full-length proteins, whereas the lower bands correspond to fragments generated by caspase-mediated cleavage, reflecting apoptosis.

Finally, we determined how NF-Y affects the promoter activity of LTR12 in the presence or absence of HDAC inhibitors. To this end, we removed the subunits alpha (NF-YA; Figure 6A and Suppl. Figure S4A) and beta (NF-YB; Suppl. Figure S4B, S4C) of NF-Y by shRNA-mediated knockdown, followed by TSA-treatment. Knockdown of each subunit was highly efficient as demonstrated by Western blot analysis and qRT-PCR (Suppl. Figure S4A-S4C).

The removal of NFY-A resulted in a decreased expression of the LTR12-driven genes *CGREF1*, *DHRS2* and *SEMA4D*, both in TSA- and in DMSO-treated cells (Figure 6A). Depletion of NF-YB also reduced the levels of LTR12-driven mRNAs, but with lower significance (Suppl. Figure S4D). LTR12-driven *TNFRSF10B* expression, in contrast, was augmented by removal of NF-YA, but not NF-YB. This is in line with a proapoptotic effect of NF-YA inactivation [50], possibly secondary to the activation of p53 [22, 49]. *TNFRSF10B* is a known p53-inducible gene [22, 23] and in this aspect differs from other LTR12-driven genes. NF-YA depletion reduced the cleavage of poly-ADP-Ribose Polymerase (PARP) and caspase 3 upon treatment with TSA (Figure 6B). Overall, we conclude that the activity of LTR12 is generally supported by NF-Y. Furthermore, TSA-mediated apoptosis is supported by NF-Y.

## DISCUSSION

LTR12, a driver of proapoptotic genes, can be induced by pharmacological HDAC inhibition. Our results show that the specific inhibition of HDACs 1, 2, and 3 is sufficient for LTR12 induction, making these HDACs primary candidates for LTR12 regulation. Strikingly, the enormous upregulation of LTR12-driven genes by HDAC inhibitors is not confined to cells from germline tumors. Instead, a large variety of cells derived from different tumor species showed LTR12 inducibility by the same agents. On the other hand, the induction by HDAC inhibitors is confined to the LTR12, derived from ERV9, but is largely absent from the LTRs from different endogenous retroviruses. Finally, NF-Y was not only found to associate with LTR12 but also to enhance its promoter activity.

### The role of HDACs 1-3 in LTR12 activation

Among the tested inhibitors, TSA, SAHA, Mocetinostat and Entinostatinduced strong LTR12 promoter activation. What these compounds have in common is their ability to inhibit the HDACs1-3. Thus, these three HDACs appear most responsible for the regulation of LTR12. HDACs 1-3 are also structurally related, and all three belong to class I of HDACs, based on their structural similarities and their homology to yeast RPD3 [38]. Members of this class of HDACs are not only targets of TSA and SAHA, but more specific additional inhibitors for these HDACs are in clinical development for cancer treatment [37]. It is conceivable that such novel HDAC inhibitors operate, at least in part, through the activation of LTR12. Of note, cancer is not the only indication for which HDAC inhibitor-based therapies are tested. Rather, HDAC inhibitors targeting class I HDACs are used in trials to treat neurodegenerative diseases as well [51]. It remains to be tested whether LTR12 expression can also be induced in degenerating neurons upon HDAC inhibitor treatment, and if so, whether this represents an obstacle or rather contributes to the beneficial effects of the drugs. In any case, LTR12-driven genes are certainly not the only ones that respond to HDAC inhibitors, although LTR12 mediates exquisitely strong gene activation by this class of drug candidates.

### A role for LTR12 activation in the anti-cancer effects of HDAC inhibitors

The mechanisms by that HDAC is exert their observed anti-cancer effects are not yet fully understood. HDACs remove acetyl groups from histone tails, and hypoacetylated chromatin is overall associated with transcriptional silencing [33, 34]. Histone deacetylation is commonly observed in human cancer and might stimulate the repression of important tumorsuppressive genes, thereby supporting tumorigenesis and possibly invasion and metastasis [39, 52, 53]. Accordingly, the inhibition of HDACs reverses these effects, and tumor proliferation is suppressed. LTR12-driven genes appear to be particularly susceptible to regulation by HDACs, not only in testicular cancer cells but in a wide variety of human cancers. This makes it plausible that the activation of LTR12-driven genes is indeed responsible for anti-tumor effects of HDAC inhibitors. At least the gene *TNFRSF10B* turned out as a mediator of apoptosis by HDAC inhibitors in our previous studies [19].

### Impact of NF-Y on LTR12 promoter activity

The identification of genomic sites of transcription factors entailed the finding that many of them bind to specific families of repetitive sequences [54]. The rationale for assaying NF-Y here was based on the finding that the trimer binds to selected HERV-LTRs, in particular LTR12 sequences [27, 54]. HDAC inhibitors increase the association of NF-Y with LTR12 (Figure 5), whereas the depletion of NF-Y attenuates both baseline and TSA-induced expression of LTR12-driven genes (Figure 6). In general, NF-Y inactivation is detrimental both for the basal and for the induced expression of LTR12-driven genes. The exception, the proapototic TNFRSF10B, shows an opposite behavior upon NF-YA *vs* NF-YB inactivation and joins a discrete set of pro-apoptotic mRNA genes which are differentially regulated upon inactivation of the two subunits [49]. What is the specific role of NF-Y in the activation of LTR12 promoters by HDAC inhibitors? Essentially, two non mutually exclusive mechanisms can be envisioned. Firstly, the effect might be entirely indirect, through hyperacetylation of neighboring nucleosomes and facilitated promoter access for transcription factors and cofactors, with the help of NF-Y. In the second scenario, HDAC inhibitors might impact directly on NF-Y, as suggested by binding of both histone acetyl transferases and HDACs to it [32, 35]. NF-YB/NF-YC show resemblance to H2B/H2A, respectively, and harbor important conserved lysine residues [25]. Since some of these are acetylated in core histones, notably in H2B, it is possible that they are targets of acetylation in NF-YB as well. Some of them are in contact with the DNA phosphate backbone, in the nucleosome and in NF-Y/CCAAT. This model would suggest that NF-Y is more stably bound to CCAAT boxes by removal of acetylation of such lysines through HDACs. However, NF-Y binding to LTR12 promoters is, if anything, increased *in vivo* after HDAC inhibition (Figure 5), while transcriptional activation is nonetheless NF-Y-dependent (Figure 6). Of note, previous *in vitro* studies did not detect increased binding of NF-Y to the TBP-2 CCAAT *in vitro* when analyzing NF-Y from SAHA-treated cells [55]. Thus, we suggest an alternative model according to that NF-Y acetylationsrepresent signals for the recruitment of cofactors, as it was shown for the RFP repressor on the TBP-2 promoter [56].

### Impact of HDAC inhibitors on cells from multiple tumor species

Interestingly, treatment with HDAC inhibitors did not only induce LTR12-regulated gene expression in testicular cancer cells, as reported previously [19, 20], but also in a set of human cancer cells derived from different tissues. SAHA (vorinostat) was approved by the US Food and Drug Administration for the treatment of cutaneous T-cell lymphoma in 2006 [45], as was romidepsin (FK228) in 2009 [57]. Both Mocetinostat (MGCD 0103) and Entinostat (MS-275) are currently undergoing clinical trials for the treatment of various cancer types, including lymphomas and solid tumors [44, 58, 59]. Our findings raise the possibility that LTR12 activation may contribute to these drug efficacies in a broader variety of cancers than hitherto anticipated. Moreover, the induction of LTR12-driven transcription may also serve as a biomarker for the efficacy of the drugs in individual tumors. Thus, HDAC inhibitors may prove useful in a broad variety of tumors. Of note, this anticipated drug efficacy may not reveal itself when tested on endogenous animal tumors. Only humans and apes show the described insertion of LTR12s upstream of proapoptotic genes [19, 20]. Thus, genetically engineered mouse (GEM) tumor models will not show a comparable gene expression pattern in their cancers upon HDAC inhibitor treatment. In contrast, when using xenograft models, derived from patients or cell lines, HDAC inhibitors may reveal their full potential for treating a broad spectrum of human cancers.

## MATERIALS AND METHODS

### Cell culture

GH (testicular cancer), H1299 (adenocarcinoma of the lung), HeLa (cervical carcinoma) and U2OS (osteosarcoma) cells were maintained in Dulbecco's Modified Eagle medium supplemented with 10% FBS. Ovcar-3 (ovarian carcinoma), K562 (chronic myelogenous leukemia) and HuT-78 (cutaneous T-cell lymphoma) cells were maintained in RPMI 1640 medium with 10% FBS. The HDAC inhibitors Trichostatin A (Sigma-Aldrich), SAHA, Mocetinostat, Entinostat, PCI-34051, Droxinostat and Tubastatin A hydrochloride (all Selleckchem) were dissolved in DMSO and added as indicated. Corresponding amounts of DMSO alone were added to controls.

### Quantitative mRNA analysis by qRT-PCR

Total RNA was isolated using TRIzol^®^ (Invitrogen), followed by reverse transcription with Moloney Murine Leukemia Virus reverse transcriptase (New England Biolabs) and a mixture of oligo(dT) and random nonamer primers. A SYBR Green master mix including Taq polymerase (Primetech) was used for real-time PCR. The primer sequences are shown in Supplemental Table S4. PCR conditions were as follows: initial denaturation 2 min at 95°C, followed by 40 cycles of 95°C for 15 sec and 60°C for 60 sec. Gene expression levels were normalized to *RPLP0*or to *RSP20* as reference genes as indicated and calculated using the 2-ΔΔCt method.

### Chromatin immunoprecipitation (ChIP)

ChIP experiments were conducted as described [60]. For the immunoprecipitation of specific target proteins, chromatin from approximately 2 × 10^6^ cells was incubated with 2 μg anti-NF-YB antibody (PAb001, Genespin, Italy) or corresponding amounts of anti-IgG antibody (ab46540, Abcam) and 30 μl protein A/G plus agarose beads (Santa Cruz). After multiple washing steps and purification, the ChIP samples were analyzed by qPCR. The primer sequences were designed according to the target sequences(Supplemental Table S3) and are shown in Supplemental Table S4. The precipitated amounts of DNA are presented as the percentage of input. As an internal negative control to exclude unspecific antibody binding, the inactive promoter region of *myoglobin* gene MB was analyzed. Cyclin B1 gene CCNB1 served as a positive control [49]. PCR conditions were as follows: initial denaturation 10 min at 95°C, followed by 40 cycles of 95°C for 15 sec and 60°C for 60 sec, and final elongation 5 min at 72°C.

### In-silico prediction

Sequence information of 22 LTR12 promoter elements (SupplementalTable S1) was retrieved from the UCSC Genome Browser (hg19) and analyzed regarding the presence of putative NF-Y binding sites by ALGGEN PROMO (8.3 version of TRANSFAC) [61, 62]. The species was defined as human only, and the dissimilarity margin was set to be equal or less than 5%. Next, LTR12 sequences were aligned using CLUSTALW2 with GAP penalties set to 25 (open), 0.20 (extension) and 5 (distances). In Supplemental Figure S2, the *insilico*-predicted NF-Y binding sites were highlighted in green to visualize their position.

### ChIP-seq data analysis

Based on the NF-YA and NF-YB binding information provided by Fleming *et al.* [27] and bed-files containing all LTR12, LTR2 or LTR7B locations in the human genome, VENN diagrams were created using the Galaxy/Cistrome platform [63]. The information was retrieved from GEO (http://www.ncbi.nlm.nih.gov/geo/), and the following data sets were used. GSM935429, GSM935433, GSM935408, GSM935508, GSM935506 and GSM935507.

### Lentiviral transduction

Scrambled control (shSC), NF-YA (shNF-YA) and NF-YB (shNF-YB) shRNAs were cloned into the pLKO.1 vector (Sigma Aldrich). Viral supernatants expressing sh-scramble (control vector), sh-NF-YA and sh-NF-YB were prepared by transfecting HEK293T packaging cells. Briefly, shRNA plasmids and second generation packaging plasmids (VSVG and pCMV-dR8.74) were transfected into HEK293T cells. Lentivirus-containing supernatants were collected 48 h after transfection, filtered and frozen until use.

Hela cells were transduced with sh-SC or sh-NF-YA or sh-NF-YB, treated with DMSO or TSA (final concentration 2μM) 54 hours after transduction, and collected at 18 hrs after treatment. Knockdown and treatment efficiency were assayed by PCR on cDNAs and by Western blot analysis on whole cell protein extracts using anti-NF-YA (Santa Cruz), anti NF-YB (GeneSpin), anti H3K9Ac (Abcam) and anti-Vinculin (Sigma) antibodies. Total RNA was prepared by Trizol extraction and reverse transcribed using the Iscript cDNA Synthesis kit (BIORAD 170-8890).

### Immunoblot analysis

After SDS-polyacrylamide gel electrophoresis and transfer on nitrocellulose, blots were incubated overnight with antibodies to PARP (Santa Cruz, sc-8007) or Caspase 3 (Cell Signaling Technologies 9662), each diluted 1:1000 in TBST with 4% BSA, followed by incubation with secondary antibodies coupled to peroxidase (1:10000) and chemiluminescent detection.

## SUPPLEMENTARY MATERIAL TABLES AND FIGURES


