# Supplementary material for: LTR12 promoter activation in a broad range of human tumor cells by HDAC inhibition
Source: Oncotarget. 2016 May 9;7(23):33484–97. doi: 10.18632/oncotarget.9255 (PMC5085097; doi:10.18632/oncotarget.9255)
Supplement: Supplementary file 1 [file oncotarget-07-33484-s001.pdf]

LTR12 promoter activation in a broad range of human tumor cells by HDAC inhibition

Supplementary Material

Table S1. LTR12 locations in the human genome.

Gene name	Gene position (hg19)	location of LTR12 (hg19)
ADH1C	chr4:100,257,649-100,273,917	chr4:100,274,696-100,275,434
GBP5	chr1:89,724,634-89,738,544	chr1:89,738,137-89,739,573
SEMA4D	chr9:91,992,152-92,094,611	chr9:92,094,404-92,095,897
TNFRSF10B	chr8:22,877,648-22,926,700	chr8:22,927,451-22,928,865
TP63	chr3:189,507,449-189,615,068	chr3:189,313,733-189,314,949
DHRS2	chr14:24,105,573-24,114,848	chr14:24,104,837-24,105,861<br>chr14:24,106,921-24,107,605
NR1H4	chr12:100,897,138-100,957,645	chr12:100,823,898-100,825,322
C9orf53	chr9:21,967,138-21,967,753	chr9:21,959,077-21,960,418
CPED1	chr7:120,628,751-120,937,498	chr7:120,699,844-120,701,243
C9orf85	chr9:74,526,423-74,588,371	chr9:74,578,275-74,579,651
CCR4	chr3:32,993,066-32,996,403	chr3:32,980,994-32,982,647
ACSBG1	chr15:78,473,097-78,527,049	chr15:78,537,615-78,539,044
KCNN3	chr1:154,669,942-154,842,754	chr1:154,650,332-154,651,788
CSF3	chr17:38,171,614-38,174,066	chr17:38,167,005-38,169,009
TMOD1	chr9:100,263,462-100,364,025	chr9:100,336,320-100,337,735
SLC36A2	chr5:150,694,539-150,727,151	chr5:150,786,356-150,787,204
IER3	chr6:30,710,976-30,712,327	chr6:30,774,508-30,775,782
PIK3C2G	chr12:18,414,474-18,801,352	chr12:18,652,900-18,654,112
CT49/ LINC01194	chr5:12,574,969-12,805,295	chr5:12,661,848-12,663,161
PTPN13	chr4:87,515,468-87,736,328	chr4:87,468,293-87,469,596
RADIL	chr7:4,836,687-4,923,335	chr7:4,832,980-4,834,366

Corresponding to Figures 4A, 4C and S2.

Table S2. *In-silico* predicted binding sites of nuclear transcription factor Y within 22 analyzed HDACi-responsive LTR12s and their relative distances.

**Site**	**Distance [nt]**	**STDEV [nt]**	**present in**
NF-Y -6	43	9	82%
NF-Y -5	28	15	73%
NF-Y -4	158	145	50%
NF-Y -3	35	23	86%
NF-Y -2	64	67	32%
NF-Y -1	37	1	91%
TATA1	83	7	95%
TATA2	353	34	64%
NF-Y +1	-/-	-/-	68%

Corresponding to Figure 4A and S2.

Table S3. Location of NF-Y bound to LTR12s in the human genome.

**Gene**	**NF-Y**	**Cell type**	**CCAAT**	**Peak coordinates**
PTPN13	YB	K	Yes	chr4:87,469,060-87,469,160
TMOD1	YB	K, G	Yes	chr9:100,336,900-100,337,400
CSF3	YB	K, G, H	Yes	chr17:38,168,300-38,168,650
	YB	K	**No**	chr17:38,167,750-38,168,050
CCR4	YB	K	Yes	chr3:32,982,100-32,982,200
SLC36A2	YB	K	Yes	chr5:150,786,750-150,787,000
KCNN2	YB	G	Yes	chr5:113,768,850-113,769,450
TNFRSF10B	YA/YB	K, G, H	Yes	chr8:22,927,750-22,928,100
PGPEP1L	YB	K, G, H	Yes	chr15:99,550,850-99,551,200
DHRS2	YB	K, G	Yes	chr14:24,105,350-24,105,700
	YB	K, G	Yes	chr14:24,106,900-24,107,250
GBP5	YB	G	Yes	chr1:89,738,550-89,738,900
C9orf53	**No**		**No**	
TENM1	**No**		**No**	

Corresponding to Figure 5.

Table S4. Oligonucleotides for quantitative real-time PCR with either cDNA or ChIP DNA as template.

Name	Template	Sequence (5'→ 3')	
RPLP0	cDNA	for	GATTGGCTACCCAACTGTTG
		rev	CAGGGGCAGCAGCCACAAA
GTAp63	cDNA	for	ATTCCGGACACCCTATCAGAG
		rev	CCCAGATATGCTGGAAAACCT
TAp63 total	cDNA	for	GTTATTACCGATCCACCATGTCC
		rev	GCGGATACAGTCCATGCTAATC
TNFRSF10B LTR12 transcript 2	cDNA	for	CCAAGTGCCTCCCTCAACTCA
		rev	CGGCGCGGCTGTACTTTCAC
TNFRSF10B total	cDNA	for	TTCTGCTTGCGCTGCACCAGG
		rev	GTGCGGCACTTCCGGCACAT
CGREF1 LTR12	cDNA	for	GAGACCAAGAACCCACCAATTC
		rev	TGGAGGGCAAAGAGGTAGAGG
ADH1C total	cDNA	for	CCACAAGTACTCACCAGCCTC
		rev	GAGGTGCAACCTCTACCTC
GBP5 total	cDNA	for	CTGCTTGACACCGAGGGC
		rev	GAGTGCCAGTGCAAAGATC
SEMA4D LTR12	cDNA	for	CACCGGGAGGAACGAACAA
		rev	CCATCAGTGTCGTCAAACATTTCA
DHRS2 LTR12	cDNA	for	CACCAAGCGGTGAGACTATCAC
		rev	CGGGCAACTGCTGACAGCATAG
APOC1 HERV-E LTR	cDNA	for	CAAGCCCTCCAGCAAGGATTCAG
		rev	GTGTGTTTCCAAACTCCTTCAG
GSDMB HERV-H LTR	cDNA	for	CTGAAATTGGCTTCTGTTTCTGAG
		rev	CCAGAATTTGAAACTCAGCC
DNAJC15 HERV-H LTR	cDNA	for	CCACCAAACAGGCTTTGT
		rev	CAGATCCGAAATGCGTAGCG
IL2RB MaLR LTR	cDNA	for	ATGTGGAACCGGCTTCCTT
		rev	GCAGATGCCCAAGAGGTAGC
HERV-K envelope [1]	cDNA	for	ATTGGCAACACCGTATTCTGCT
		rev	CAGTCAAAATATGGACGGATGGT
HERV-W envelope [2]	cDNA	for	ATGGAGCCCAAGATGCAG
		rev	AGATCGTGGGCTAGCAG
RSP20	cDNA	for	GCGCCTCTTATCAAGTCAGC
		rev	CGGAAAAACACCCGTGGAG
NF-YA	cDNA	for	TCAATTCAGGAGGGATGGTC
		rev	GCCGAGACTCATGCAGGTAT
NF-YB	cDNA	for	AGGTGCCATCAAGAGAAACG
		rev	TGTTGTTGACCGTCTGTGGT
CCNB2	cDNA	for	AGTTCCAGTTCAACCCACCA
		rev	GCAGAGCAAGGCATCAGAAA
PHGDH	cDNA	for	GCGTGTGGTGAACTGTGC
		rev	AGGTGGGGACAGCTGATG
Myoglobin	ChIP DNA	for	CTCATGATGCCCCTTCTTCT
		rev	GAAGGCGTCTGAGGACTTAAA
CCNB1 [3]	ChIP DNA	for	CCCCGCCCCTCTCGAAC

		rev	TTAAACCCCGCACTGCTCCC
DHRS2 LTR12	ChIP DNA	for	GGACCAATCAGCTCTCC
		rev	GAACCAGAGCAGGTTGCTGC
PGPEP1L LTR12	ChIP DNA	for	CACCCACATCCTGCTGATT
		rev	TCCAGCTCCAGGATTGTAAAC
TNFRSF10B LTR12	ChIP DNA	for	CGCTGATTGGTGGTTTACAATC
		rev	GAATGCACCAATTGACACTC
GTAp63 LTR12	ChIP DNA	for	CAGACCACTCGGCTCTACCAATC
		rev	GTGTGCACCCAAAGAGTGAG

Corresponding to Figures 1, 2, 3, 5, 6, S1 and S4B,C.

References

1. Wang-Johanning F, Frost AR, Jian B, Epp L, Lu DW and Johanning GL. Quantitation of HERV-K env gene expression and splicing in human breast cancer. Oncogene. 2003; 22(10):1528-1535.
2. Ruebner M, Strissel PL, Ekici AB, Stiegler E, Dammer U, Goecke TW, Faschingbauer F, Fahlbusch FB, Beckmann MW and Strick R. Reduced syncytin-1 expression levels in placental syndromes correlates with epigenetic hypermethylation of the ERVW-1 promoter region. PloS one. 2013; 8(2):e56145.
3. Benatti P, Dolfini D, Vigano A, Ravo M, Weisz A and Imbriano C. Specific inhibition of NF-Y subunits triggers different cell proliferation defects. Nucleic acids research. 2011; 39(13):5356-5368.

LTR12 induction by HDAC inhibitors in cell lines derived from various tumor species.

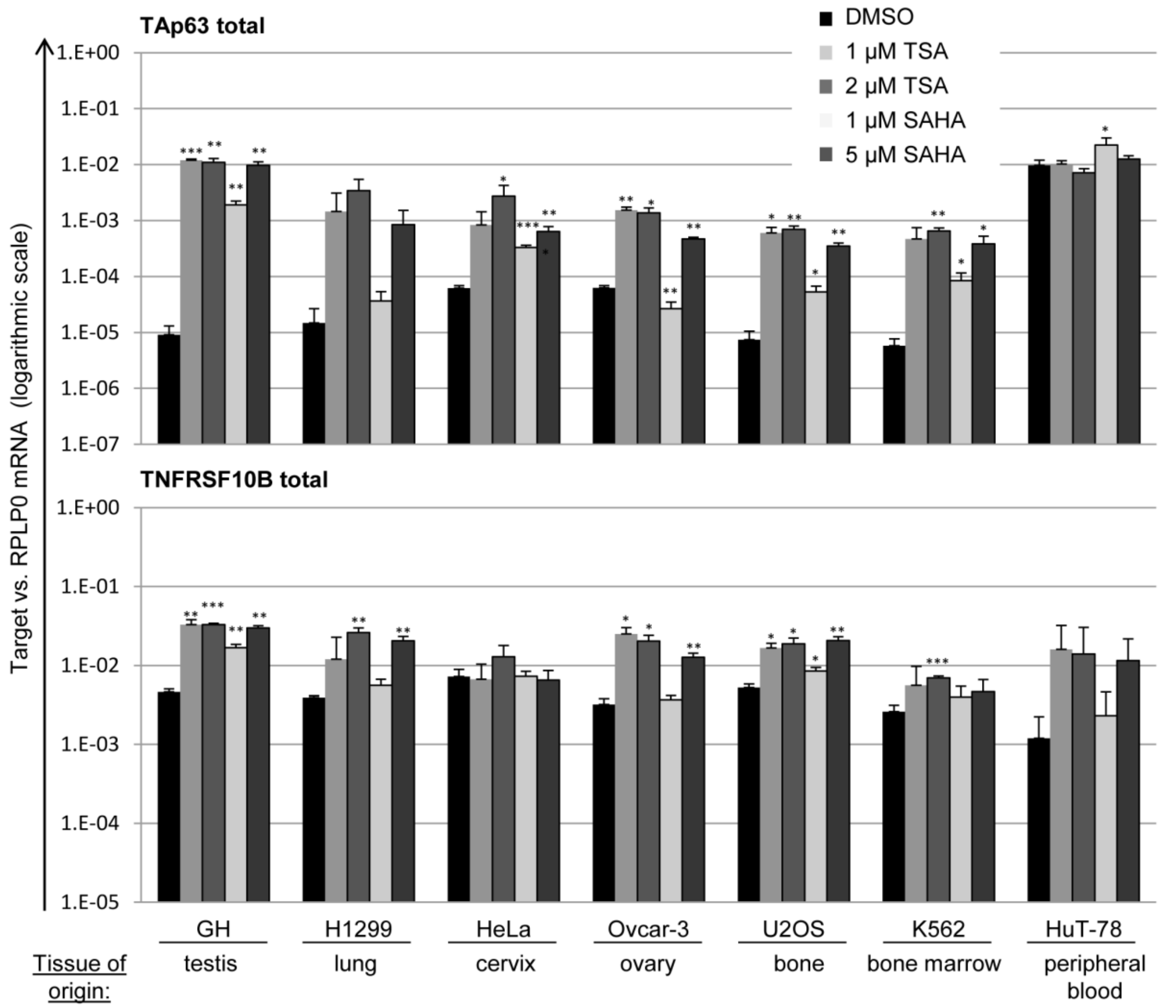

Corresponding to Figure 2.

Figure S1: Total levels of mRNA corresponding to TAp63 and TNFRSF10B

Human cell lines derived from different tissues were treated with with HDAC inhibitors Trichostatin A (TSA) or suberoylanilide hydroxamic acid (SAHA). Cells were treated with increasing concentrations of each inhibitor (1 µM, 2 µM for TSA and 1 µM, 5 µM for SAHA) for

18 h. After this incubation time, relative gene expression was assessed by qRT-PCR. The transcription levels corresponding to *TAp63* [A] and *TNFRSF10B* [B] are depicted. The primers were chosen to amplify all such mRNAs, regardless of whether it had the transcription start site at the LTR12 promoters or at the standard promoters that were previously described (cf. Gene IDs 8626 and 8795 and primer sequences shown in Supplemental Table S4). In comparison to the LTR12-driven isoforms, the increase in gene transcription was less strong for the “total” transcripts, which include mRNAs originating from both transcription start sites. mRNA levels were normalized to *RPLP0*. SD (n=3). * = $p<0.05$, ** = $p<0.01$, *** = $p<0.001$.

Corresponding to Figure 2.

```
ADH1C_LTR12      ------------------------------------------------------------------------------------------ 1
GBP5_LTR12C      TGAGAGGTGACGGCGTGCTGGCAGCCCTTG-CAGCCCTCCCTCGCTCTCGGTGCCTCCTCTGCCTGGGCACCCACTCTGGCGGCACTTGA 89
SEMA4D_LTR12C    TGAGAGGTGACAGCGTGCTAGCAGTCCTCA-GAGCCCTCGCTTGCTCTCGGCACCTCCCCTGCCTGGGCTCCCACTTTGGCGGCATTTGA 89
TNFRSF10B_LTR12  TGAGAGGTGACAGCTTGCTGGCAGTCCTCAACAGCCCTCGCTCGCTCTCGGCGCCTCCTCTGCCTGGGCTCCCACTTTGGCGGCACTTGA 90
TP63_LTR12C      -GAGAGGTGACAGCGTGCTGGCAGTCCTCA-CAGCCCTCGCTCGCTCTGGGCGCCTCCTCTGCCTGGGCTCCCACTTTGGTGGCACTTGA 88
DHRS2_LTR12D_1   -GAGAGGTGACAATATGCTAGCAG----------CCCTTGCTCACTCTTGGTGCCTCCTCGTCCTCCACATCCACTCTGGCTGCACTTGA 79
DHRS2_LTR12D_2   ------------------------------------------------------------------------------------------ 1
NR1H4_LTR12C     -GAGAGGTGACAGCGTGCTAGCAGTCCTCA-CAGCCCTCGCTCGCTCTCGGCGCCTCCTCTGCCTGGGCTCCCACTTTGGCGGCACTTGA 88
C9orf53_LTR12CD  -GAGAGGTGACAGCATGCTGGCAGTCCTCA-GAGCCCTCGCTTGC-------ACCTCCCCTGCCTGGGCTCCCACTTTGGCGGCATTTGA 81
CPED1_LTR12C     -GAGAGGTGACAGCATGCTGGCAGTCCTCA-CAGCCTTCGCTCGCTCTCGGCGCCTCCTCTGCCTGGGCTCCCACTTTGGCCGCACTTCA 88
C9orf85_LTR12C   -GAGAGGTGACGGCGTGCTGGCAGTCCTCA-CAGCCCTCGCTCGCTCTCGGTGCCTCCTCTGCCTGGGCTCCCACTTTGGCGGCACTTGA 88
CCR4_LTR12C      -GAGAGGTGACAGCGTGCTGGCAGTCCTCA-GAGCCCTCGCTTGCTCTCGGCGCCTCCTCTGCCTGGGTTCCCACTTTGGCGGCATTTGA 88
ACSBG1_LTR12C    -GAGAGGTGACAGCGTGCTGGCAGTCCTCA-CAGCCCTTGCTCGCTCTCGGCGCCTCCTCTGGCTGGGCTACCACTCCGGCGGGACTTGA 88
KCNN3_LTR12C     -GAGAGATGACAGCGTGCTGGCAGTCCTCA-CAGCCCTCGCTCGCTCTCGGCGTCTCCTCTGCCTGGGCTCCCACTTTGGCGGCACTTGA 88
CSF3_LTR12CD     -GAGAGGTGACAGCATGCCGGCAGTCCTCA-CAGCCCTCGTTCGCTCTCGGCGCCTCCTCTGCCTGGGCTCCCACTTCGGTGGCACTTGA 88
TMOD1_LTR12EB    -GAGAGGTGACAGCATGCTGGCAGCCCTCA-CAGCCCTCCCTCGCTCTCGGCGCCTCCTCAGCCTTGGCGCCCACTCTGGCCGCGCTTGT 88
SLC36A2_LTR12    ------------------------------------------------------------------------------------------ 1
IER3_LTR12C      TGAGAGGTGACAGCGTGCTGGCAGTCCTCA-GAGCCCTCGCTTGCTCTTGCCACCTCCTCTACCTGGGCTCCCACTTTGGCGGCATTTGA 89
PIK3C2G_LTR12C   -GAGAGGTGACAGCGTGCTGGCAGTCCTCA-CAGCCCTCGCTCGCTCTCGGCGCCTCCTCTGCCTGGGCTCCCACTTTGGCGGCACTTGA 88
CT49_LTR12E      -GAGAGCTGACAACATGCTAGCAG----------CCCTCGCTCGCTCTTGGTGCCTCCTCGGCCTCGGCGTCCACTCTGGCTGCGCTTGA 79
PTPN13_LTR12EC   -GAGAGGTGACAGCTTGCTGGCAGTTCTCA-CAGCCCTCGCTTCCTCTCGGCGCCTCCCCTGCCTGGGTTCCCACTTTGGCGGCACCTGA 88
RADIL_LTR12C     -GAGAAGTGACAGCCTGCTGGCAGTCCTCA-CAGCCCTCGCTCGCTCTTGGCGCCTCCTCTGCCTGGGCTCCCACTTTGGCAGCACTTGA 88
Clustal Consens                                                                                             1

ADH1C_LTR12      ------------------------------------------------------------------------------------------ 1
GBP5_LTR12C      GGAGCCCTTCAGCCCTCCGCTGCACTGTGGGAGCCCCTTCCT-GGGCTGGCCGAGGCCGGAGCTGGCTCCCTCAGCTTGCTGGGAGGTGT 178
SEMA4D_LTR12C    GGAGCACTTCAGTCCCCCACTGCACTGTGGGAGCCCCTTTCT-GGGCTGGCCAAGGCCGGAGCCCACTCCCCCAGTTTGTAGGGAGGTGT 178
TNFRSF10B_LTR12  GGAGCCCTTCAGCCCACCGCTGCACTGTGGGAGCCCTTTTCT-GGGCTGGCCAAGGCTGGAGCCCGCTCCCTCAGCTTGCACGGAGGTGT 179
TP63_LTR12C      GGAGCCCTTCAGCCCACTGCTGCACTGTGGGAGCCTCTT-CT-GGGCTGGCCAAGGCCGGAGCCCGCTCCCTCAGCTTGCAGGGAGGTGT 176
DHRS2_LTR12D_1   GGAGCCCTTCAGCCCACCACTGCACTGTGGGGGCCCCTCTCTGGGGCTGGCCAAGGCCGAAGCCAGCTCCCTCTGCTCGCCAGGAGGTGT 169
DHRS2_LTR12D_2   ------------------------------------------------------------------------------------------ 1
NR1H4_LTR12C     GGAGCCCTTCAGCCCGCCGCTGCACTGGGGGAGCCCCTTTCT-GGGCTGGCCAAGGCTGGAGCCGGCTCCCTCAGCTTGCAGGGAGGGGT 177
C9orf53_LTR12CD  GGAGCCCTTCAGCCCCCACCTGCACTGTGGGAGCCCCTTTCT-GGGCTGGCCAAGGCTGGAGCCCACTCCCTCAGCTTGCAGGGAGGTGT 170
CPED1_LTR12C     GGAGCCCTTCAGCCCACTGCTGCACTGTGGGAGCCCCTTTCT-GGGCTGGCCAAGACCGGAGCCGTCTCCCTCAGCTTGCAGGGAGGTGT 177
C9orf85_LTR12C   GGAGCCCTTCAGCCCACCGCTGCATTGTGGTAGCCCCTTTCT-GGGCTGGCCAAGTCTGGAGCCAGCTCCCGCAGCTTGCAGGGAGGTGT 177
CCR4_LTR12C      GGAGCCCTTCGGCCCGCCGCTGCACTGTGGGAGCCCCATTCT-GGGCTGGCCAAGGCCAGAGCCGGCTCCCTCAGCTTGCAAGGAGGTGT 177
ACSBG1_LTR12C    GGAGCCCTTCAGCCCACCGCTGCACTGTGGGAGCCCCTTTCT-GGGCTGGCCAAGGCCGGAGCCGGCTCCCTCAGCTTGTCGGGAGGTGT 177
KCNN3_LTR12C     GGAGCACTTCAGCCCACTGCTGCACTGTGGGAGCCCCTTTCT-GGGCTGGCCGAGGCTGGAGCCGGCTCCCTCAGCTTGCAGGGAGGTGT 177
CSF3_LTR12CD     GGAGCCCTTCAGCCCACCGCTGCACTGTGGGAGCCCCTTTCT-GGGCTGGCCAAGGCCAGAGCCGGCTCCCTCAGCTTGCAGGGAGGTGT 177
TMOD1_LTR12EB    GGAGCCCTTCAGCCCGCCGCTGCACTGTGGGAGCCCCTTTCT-GGGCTGGGCAAGGCCAGAGCCGGCTCCCTCAGCTTGCGGGGAGGTGT 177
SLC36A2_LTR12    ------------------------------------------------------------------------------------------ 1
IER3_LTR12C      CGAGCCCTTCAGCCCACCACTGCACTGTGGGAACCCCTTTCT-GGGCTGGCCAAGGCTGGAGCCCACTCCCTCAGCTTGCAGGGAGGTGT 178
PIK3C2G_LTR12C   GGAGCCCTTCAGCCGGCCGCTGCTGTGTGGGAGCCCCTTTCT-GGGCTGGCCAAGGCCGGAGCTGGCTCCCTCAGCTTGCGGGGAGTTAT 177
CT49_LTR12E      GAAGCCCTTCAGCCCACCGCTGCACTGTGGGAGCCTTCTCT--GGGCTGGCTGAGGCTGGAGCCGGCTCCCTCTGCTTGCAGGGAGGTGT 167
PTPN13_LTR12EC   GGAGCCCTTCAGCCCGCC-CTGCACTGTGGGATCCCCTTTCT-GGGCTGGCCAAGGCCGGAGCCGGCTCCCTCAGCTTGCCAGGAGGTGT 176
RADIL_LTR12C     GGAGCCCTTCAGCCCGCCACTGCACTGTGGGAGCCCCTTTCA-GGGCTGGCCAAGGCCGGAGCCGGCTCCCTCAGCTTGCGGGGAGGTGT 177
Clustal Consens                                                                                             1

ADH1C_LTR12      ------------------------------------------------------------------------------------------ 1
GBP5_LTR12C      GGAGGGAGAGGCAGGGGTGGGAACTGGGGCTGCGCCCG--GTGCTTGAGGGCCAGCGCGAGTTCTGGGTGGGCTTGGGCTCTGAGGGACC 266
SEMA4D_LTR12C    GGAGGGAGAGGCAGGAGCGGGAACAGGGGCTGTGTGCG--GCACTTGCGGGCCAGCTGGAGTTCCGGGTGGGCGTGGGCTTGGTGGGCCC 266
TNFRSF10B_LTR12  GGAGGGAGAGGCGCGAGCGGGAACCGGGGCTGCGCGCGCGGCACTTGCGGGCCAGCTGGAGTTCCGGGTGGGCGTGGGCTCGGCGGGCCC 269
TP63_LTR12C      GGAAAGAGAGGTGGGAGCGGGAACTGGGGCTGCGCGCG--GCGCTTCCGGGCCAGCTGGAGTTCCGGGTGGGCGTGGGCTTGGCGGGCTC 264
DHRS2_LTR12D_1   GGAGGGAGAGGCGCCGGCAGGAGCCCACACTGTACAGG--GCACTCACCGGCCAGCAGGGGCTCC--GTGGGCC---------------- 239
DHRS2_LTR12D_2   ------------------------------------------------------------------------------------------ 1
NR1H4_LTR12C     GGAGAGAGAGGCGCGAGCGGGAACCGGGGCTGCGTGCG--GCGCTTGCGGGCCAGCTGGAGTTCCGGGTGGGCGTGGGCTTGGCGGGCCC 265
C9orf53_LTR12CD  GGAGGGAGAGGCGCGAGCGGGAACCGGGGCTGCGTGCG--GCGCTTGCGGGCCAGCTGGAGTTCCGGGTGGGCGTGGGCTTGGCGG-CCC 257
CPED1_LTR12C     GGAGGGAGAGGCGCGAGCGGGAACCCGGGCTGCGCGCG--GCGCTTGCGGGCCAGCTGGAGTTCCGGGTGGGCGTGGGCTTGGCGGGCCC 265
C9orf85_LTR12C   GGAGGGAGAGGCGTGAGCGGGAACCCGGGCTGCGCGAG--GCGCTTGCGGGCCAGCTGGAGTTCCGGGTGGGCGTGGGCTTGGCGGGCCC 265
CCR4_LTR12C      GGAGGGAGAGGCGCGAGCAGGAACCAGGGCTGCGAGCA--GCGCTTGCGGGCCAGCTGGAGTTCTGGGTGGGCGTGGGCTTGGCTGACCC 265
ACSBG1_LTR12C    GGAAGGAGAGGCGCCAGCGGGAACCGGGGCTACG-GCGAGGCGCTTGCAGGCCAGCTGTAGTTCCGGGTGGGCGTGGGCTTGGCGGGCCC 266
KCNN3_LTR12C     GGAGGGAGAGGTGCGAGTGGGAACCCGGGCTGTGCGCG--GCGCTTGTGGGCCAGCTGGAGTTCCGGGTGGGCGTGGGCTTGGTGGGCCC 265
CSF3_LTR12CD     GGAGGGAGAGGCTCAAGCAGGAACCGGGGCTGCGCGCG--GCGCTTGCGGGCCAGCTGGAGTTCCGGGTGGGCGTGGGCTTGGCGGGCCC 265
TMOD1_LTR12EB    GGAGGGAGAGGCGCCGGCGGGAACCAGGGCTGTGCGCG--GCGCTTGCGGGCCAGCGCGTGTTCCGGGTGGGCGTGCTCTCGGCGGGCCC 265
SLC36A2_LTR12    ------------------------------------------------------------------------------------------ 1
IER3_LTR12C      GGAGGGAGAGGTGCGAGCGGGAACCGGGGCTGCGAGCG--GCGCTTGGGCGCCAGCGGGAGTTCCGGGTGGGCGTGGGCTTGGAGGGCCC 266
PIK3C2G_LTR12C   GGAGGGAGAGGCGCGGCCGGGAACCGGG-CTGCGCCCA--GTGCTTGCCGGCCGGCTGGAGTTCCAGGTGGGCGTGGGCTTGGCGGGCCC 264
CT49_LTR12E      GGAGGGAGAGGTGCGGGCGGGAACCGGGGGTGTGTGCG--GTGCTCATGGTCCAGTGTGAGTTCCCAGTGGGCCCAGGCTTGGCAGGCCC 255
PTPN13_LTR12EC   GGAGGGAGAGGCGCTGGGGGAACCAGGACTGCGCGAG------------------------TTCCGGGTGGGCGTGGGCTCTGCGGACCC 243
RADIL_LTR12C     GGAGGGAGAGGCGCG-GTGGGAACCAGGGCTGCACGCG--GTGCTTGCGGGCCAGCGCGAGTTCCGGGTGGGCGTGGGCTCTGTGGGCCC 264
Clustal Consens                                                                                             1


ADH1C_LTR12      ------------------------------------------------------------------------------------------ 1
GBP5_LTR12C      TGCACTCGGAGC------------------GGCCCCAGGCAGTGAGGGGCTTAGCACCTGGGCCAGCAGCTGCTG--------------- 323
SEMA4D_LTR12C    CGCACTCGGAGCAGCCAGCCAGCCCTTCT-GGCCCCGGGCAATGGGGACTTAGCACCCGGGCCAGTGGCTGCGGAGGGTGTACTGAGTC 355
TNFRSF10B_LTR12  TGCACTCGGAGCAGCCGGCCGGCCCTGCC-GGCCCCGGGCAATGAGGGGTTTAGCACCCGGGCCAGCGGCTGCGGAGGGTGTACTGGGTC 358
TP63_LTR12C      TGCACTCGGAACAGCCGGCCGGCCCCGCC-GGCCCCAGGCAATGAGGGGCTTAGCACCCGGGCCAGCAGCTGTGGAGGGTGTACTGGGTC 353
DHRS2_LTR12D_1   ----------------GGCCGGTGCCAGC-TGGGCCTGACTGGGGATG-------------------------------------AGCTC 276
DHRS2_LTR12D_2   ------------------------------------------------------------------------------------------ 1
NR1H4_LTR12C     GGCACTCGGAGCAGCCGGCCAGCCCTGCC-GGCCCCGGGCAATGAGGGGCTTAGCACCCGGGCCAGCAGCTGCGGAGGGTGTACTGGGTC 354
C9orf53_LTR12CD  CGCACTCAGAGCAGCCAGCCAGCCCCGCT-GGCCCCGAGGAATGAGGGACTTAGCACCCGGGCCAGTGGCTGCGGAGAGTGTACTGGATC 346
CPED1_LTR12C     TGCACTCGGAGCAGCCGGCCGGCCCTGCC-GGCCC-GGGCAATGAGGGGCTTAGCACCCGGGCCAGCGGCTGCGGAGGGTGTGCTGGGTC 353
C9orf85_LTR12C   TGCACTCAGATTAGCTGGCCGGCCCTGCC--GCCCCGGGCAATGAGGGGCTTAGCACCCAGGCCAGCGGCTGCAGAGGGTGTACTGGGTC 353
CCR4_LTR12C      CGCACTTGGAGAAGCCCGCCAGCCCTGTC-GGCCCCGGGCAGTGAGGGGCTTAGCACCCGGGCCAGCGGCTGCGGAGGGTGTACTGGGTC 354
ACSBG1_LTR12C    TGCACTCAGAGCAGCCGGCCGGCCCTGCC-GGCCCCGGGCAATGAGGGGCTTAGCACCCGGGCCAGCAGCTGCAGAGGGTGTACTGGGTC 355
KCNN3_LTR12C     CGCACTCGGAGCAGCCGGCCGGCCCTGCC-AGCCCCGGGCAATGAGGGGCTTAGCACCCGGGCCAGCGGCTGCGGAGGGTGTACTGGGTC 354
CSF3_LTR12CD     CGCACTCGGAGCAGCGGGCCAGCCCTGCCAGGCCCCGGGCAATGAGAGGCTTAGCACCCGGGCCAGCGGCTGCGGAGGGTGTACTGGGTG 355
TMOD1_LTR12EB    CGCACTCGGAGCGGCCGGCCGACCCCGCC-GGCTCCGGGCAGTGAGGGGCTTAGCACCTGGGCCAGCAGCT------------------- 335
SLC36A2_LTR12    ------------------------------------------------------------------------------------------ 1
IER3_LTR12C      CGCACTCAGAGCAGCCGGCCAGCCCTGCC-GGCCCCGGGCAATGAGGGACTTAGCACCCGGGCCAGTGGCTGCAGAGGGTGTACTGGGTC 355
PIK3C2G_LTR12C   CGCACTCGGAGCGGCCGGCCGGCCCTGCC-GGCCC-GGGCAATGAGGAGCTTAGCACCTTGGCCAGCGGCGGCGGAGGGTGTGCTGGGTC 352
CT49_LTR12E      TGCACTTGGAGTGGGCGGCCAGCGCCGCC-AGCCCTGGGCAGTGAGGGGCTTAGCACCCAGGCCAGCAGCTGCAGAAGGTGCACCGGGTC 344
PTPN13_LTR12EC   -GCACTCCCAGCAGTCGGCCGGCCCCACC--GCCCGGGGCAGTGAGGGGTTTAGCACCTGGGCCAGCAACT------------------- 311
RADIL_LTR12C     CGCACTCGGAGCCGCCGGCTAGCCCCGCC--AGCCTGGGCAGTGAGGGGCTTAGCACCTGGACCAGCAGCT------------------- 333
Clustal Consens                                                                                             1
```

```
ADH1C_LTR12        ------------------------------------------------------------------------------------------ 1
GBP5_LTR12C        --------------------------------TGCTCCACTTCTTGCAGGGCCTTAGCTGCCTCCCGGTGGGGCAGGGCTGGGGACCTGC 381
SEMA4D_LTR12C      CCCCAGCAGTGCTGGCCCACCGGCGCTGTGCTCG----ATTTCTCACCGGGCCTTGACTGCCTTCCCACGGGGCAGGGCTCGGGACCTGC 441
TNFRSF10B_LTR12    CCCCAGCAGTGCCGGCCCACTGGCGCTGCGCTTG----ATTTCTCGCCGGGCCTTAGCTGCCTTCCCGACGGGCAGGGCTCG-------- 436
TP63_LTR12C        CCCCAGCAGTGCCAGCCCACCAGCGCTGCGCTCG----ATTTCTCACCGGGCCTTAGCTGCCTTCCCGTGGGGCAGGGCTCGGGACCTGC 439
DHRS2_LTR12D_1     CCTCTGGGCTGCTGGAGTGCCTGGGCTAGG------------------------------------------------------------ 306
DHRS2_LTR12D_2     ------------------------------------------------------------------------------------------ 1
NR1H4_LTR12C       CCCCAGCAGTGCCAGCCCACCGGCGCTGCTCTCG----ATTTCTCACCGGGCCTTAGCTGCCTTCCCGCGGGACAGGGCTCGGGACCTGC 440
C9orf53_LTR12CD    CCCCAGCAGTGCCGGCCCACCGGCGCTGTGCTCG----ATTTCTCGCCGGGCCTTAGCTGCCTTCCCGCGGGGCAGGGCTTGGGACCTGC 432
CPED1_LTR12C       CCCCAGCAGTGCCAGCCCAGTGGCGCTGTGCTCG----ATTTCTCGCCGGACCT----------------------------------GC 405
C9orf85_LTR12C     CCCCAGCAGTGCCAGCCCACTGGCGCTGCGCTTG----ATTTCTCCCCGGGCCTTAGCTGCCTTCCTGCGGGGCAGAGCTCGGGACCTGC 439
CCR4_LTR12C        CCCCAGCAGTGCCAGCCCACAGGCGCTGCGCTCA----ATTTCTCGCTGGGCCTTAGCTGCCTTCCCACGGGGCAGGCCTCGGGAC-TGC 439
ACSBG1_LTR12C      CCCCAGCAGTGCCAGCCCACCGGCGCTGCGCTCG----ATTTCTCGCTGGGCCTTAGCTGCCTTCCCGTGGGGCAGGGCTCGGGACCTGC 441
KCNN3_LTR12C       CCCCAGCAGTGCCAGCCCGCTGGCGCTGCGCTCG----ATTTCTCACCAGGCCTTAGCTGCCTTCCCATGGGGCAGGGCTCGGGACCGGC 440
CSF3_LTR12CD       CCCCAGCAGTGCCAGCCCGCCGGCGCTGTGCTCGCTCGATTTCTCACTGGGCCTTAGCAGCCTTCCCGCGGGGCAGGGCTCGGGACCTGC 445
TMOD1_LTR12EB      ------------------------GCTGTGCTCG----ACTTCTCGCCGGGCCTTAGCTGCCTCCCCACGGGGCAGGGCTCGGGACCTGC 397
SLC36A2_LTR12      ------------------------------------------------------------------------------------------ 1
IER3_LTR12C        GCCCAGCAGTGCCAGCCCACCGGCGGTGCGCTCG----ATTTCTCACCGAGCCTTAGCTGCCTTCCCGCGGGGCAGGGCTCGGGACCTGC 441
PIK3C2G_LTR12C     CCCCAGCAGTGCCGGCCCACCAGCGCTGCGCTGG----ATTTCTCGCCGGGCCTTAGCTGCCTTCCCGCGGGGCAAGGCTCGGGACCTGC 438
CT49_LTR12E        CCCCAGCACTGCTGGCCTGCCCATGCCATGCTCG----AATTCTCGCTGGGCCTCAGTTGCATCCCCGTGTGGCAGGGCTCAGGACCTGC 430
PTPN13_LTR12EC     ------------------------GCTGTGCTCA--AAATTTCTCGCCGGGCCTTAGTTGCCTTCCCGCAGGGCAGGGCTCAGGACCTGC 375
RADIL_LTR12C       ------------------------GCTGTGCTCG----ATTTCTCACTGGGCCTTAGCTGCCTTCCTGCGGGGCAGGGCTGGGGACCTGC 395
Clustal Consens                                                                                               1

ADH1C_LTR12        ------------------------------------------------------------------------------------------ 1
GBP5_LTR12C        AGCCTGCCATGCCTGAGCCTCCTCCCCTCTCCCGCTCCCCACCTCACCCCGCCATGGGTTCCTGTGCTGCCTGAGCCTCCCCAACCTGTG 471
SEMA4D_LTR12C      AGCCCGCCATGCCTGAGCCTCCCACC--CACTCCATGGGCTCCT-------------------GTGCGGCCCGAGCCTCCCCGACGAGAG 510
TNFRSF10B_LTR12    GGACCGCCATGCCTGAGCCTCTCACC--CCCTCCGTGGGCTCCT-------------------GTGCAGCCGGAGCCTCCCCGACGAGCA 505
TP63_LTR12C        AGCCCGCCATGCCTGAGCCTCCCACC--CGCTCTGTGGGCTCCT-------------------GTGTGGCCGGAGCCTCCTCGACAAGCG 508
DHRS2_LTR12D_1     ------------------------------TGCCGCAAAGTCCT-------------------GCA------------------------ 323
DHRS2_LTR12D_2     ------------------------------------------------------------------------------------------ 1
NR1H4_LTR12C       AGCCCGCCATGCTTGAGCCTCCCACC--CCCTCCATGGGCTCCT-------------------GTGCGGCCTGAGCCTCCCCGATGAGCG 509
C9orf53_LTR12CD    AGCCCGCCATGCCTGAGCCTCCCACC--CACTCCATGGGCTCCT-------------------GTGCTGCCCGAGCCTCCCCGACGAGCA 501
CPED1_LTR12C       AGCCCACCATGCCTGAGCCTCCCACC--CCCTCCGTGGGCTCCT-------------------GTGCGGCCGGAGCCTCCCTGATGAGCG 474
C9orf85_LTR12C     AGTCCGCCATGCCTGGGCCTCCCACC--C-CTCCCTGGACTCCT-------------------GTGCAACCTGAGCCTCCCCTACCAGCG 507
CCR4_LTR12C        AGCCCACCATGCCTGAGCCTTCCCCC--GCCTCCGTGGGTTCCT-------------------GTGCAGCCTGAGCCTCCCCGACGAATG 508
ACSBG1_LTR12C      AGCCCGCCATGCCTGAGCCTCCCACC--CCCTCCGTGAGCTTCT-------------------GTGCGGCCCGAGGCTCTTCGACGAGCG 510
KCNN3_LTR12C       AGCCCATCATGCCTGAGCCTCCCACC--CCCTCCGTGGGCTCCCAAGCCTCCCCG--------GTGTGTCCGGAGCCTCCCCGATGAGCA 520
CSF3_LTR12CD       AGCCCGCCATGCCTGAGCCTCCC-------CTCCATGGGCTCCT-------------------GTGCGGCCCGAGCCTCCCCGACGAGCA 509
TMOD1_LTR12EB      AGCCCGCCATGCCTGAGCTTCCCCCA----CGCCGTGGTCTCCT-------------------GAGCGGCCCGAACCTCCCCGACAAGCG 464
SLC36A2_LTR12      ------------------------------------------------------------------------------------------ 1
IER3_LTR12C        AGCCCGCCATGCCTGAGCCTCCCACC--CACTCAAAGGGCTCCT-------------------GTGCGGCCCGAGCCTCCCCGACGAGCA 510
PIK3C2G_LTR12C     AGCCAGCCATGCCTGAGCCTCCCCAA--GCCTCCATGTGCTCCT-------------------GTGCAGCC-GAGCCTCCCCTACGAGCG 506
CT49_LTR12E        AGCCCGCCATGCCCAAGCC---CCCC--ACTGTGGTGGGCTCCT-------------------GCGTGGCCTGAGCCTCCCTGATGGGCG 496
PTPN13_LTR12EC     AGCCCGCCATGCCTGAGCCTCCCCCG--CGCTCCCTGGGCTCCT-------------------GTGCCG-CGGAGCCTCCCCTAAGAGCG 443
RADIL_LTR12C       AGCCCGCCATGCCTGAGCCTCCCCCCTCCCCTCCGTGGGTTCCT-------------------GCGCAGTCCGAGCCTCCCCAACGAGTG 466
Clustal Consens                                                                                               1

ADH1C_LTR12        ------------------------------------------------------------------------------------------ 1
GBP5_LTR12C        CTGACCCCTGCTCCACAGCACCCTGTCCCATCCATGGCCCAAGGACCGAGGAGTGCC---AGTGCATGGAGCAGGA-------------C 545
SEMA4D_LTR12C      CCACCCCCTGCTCCACGGCGCCCAGTCCCATCGACCACCCAAGGGCTGAGGAATGCG---AGCGCACGGCACAGGA-------------C 584
TNFRSF10B_LTR12    CCTCCCCCTGCTCCAGGGCGCCCAGTCCCATCGACCACCCAAGGGCTGAGGAGTGCG---GGCGCACGGCGCGGGAGCACGGCGCGGGAC 592
TP63_LTR12C        CCACCCCCTGCTCCACGGCGCCCAGTCCCATCGACCACCCAAGGGCTGAAGAGTGCA---GGCGCATGGCGCGGGA-------------C 582
DHRS2_LTR12D_1     -------------------------------------------------------------GTGTGTGCCATTGAG-------------- 338
DHRS2_LTR12D_2     ------------------------------------------------------------------------------------------ 1
NR1H4_LTR12C       CCGCCCCCTGCTCCACGGCACCCAGTCCCATCAACCACCCAAGGGCTGAGGAGTGTG---GGCACACGGCACGGGA-------------C 583
C9orf53_LTR12CD    CCACCCCCTGCTCCACGGCGCCCAGTCCCAACGACCACCCAAGGGCTGACGAATGCG---AGCGCACGGCGCAGGA-------------C 575
CPED1_LTR12C       CCGCCCCTTGCTCCACGGCGCCCAGTCCCATCGACCACCCAAGGGCTGAGGAGTGCG---GGCGCACGGCGCGGGA-------------C 548
C9orf85_LTR12C     CCACCCCCTGCTCCACGGAGCCCAGTCCCATCAACCACCCAAGGGCTGAGGAGTGCG---GGCGCACGGCACGGGA-------------C 581
CCR4_LTR12C        CCGCCCCCTGCTCCAGGGCGCCCAGTCCCATCGACCGCCCAAAGGGTGAGGAGTGCG---AGCGCATGGCGCGGGA-------------C 582
ACSBG1_LTR12C      CCACCCCCTGCTCCACAGCGCCCAGTCCCATCGAACACCCAAGGGCTGAGGAGTGCA---GGCGCACAGCGCGGGA-------------C 584
KCNN3_LTR12C       CCGCCCCCTGCTCCATGGTGCCCAGTTCCATTGACCACCCAAGGGCTGGGGAGTGTG---GGCGCACAGCGTGGGA-------------C 594
CSF3_LTR12CD       CCACCCCCTGCTCCACAGCGCCCAGTCCCATCGACCACGCAAGGGCTGAGAAGTGCG---GGCGCACGGCACCGGG------------AC 584
TMOD1_LTR12EB      CCGCCCCCTGCTCCACGGCGCCCAGTCCCATCAACCACCCAAGGGCTGAGGAGTGCG---GGCGCAGGGCGCGGGA-------------C 538
SLC36A2_LTR12      ------------------------------------------------------------------------------------------ 1
IER3_LTR12C        CCACCCCCTGCTCCACGACGCCCAGTCCCATCGACCACCCAAGGGCTGAGGAGTGCA---AGCACACGGCACAGGA-------------C 584
PIK3C2G_LTR12C     CGGCCCCCTGCTCCACGGCGCCCAGTCCCATCGACCACCTAAGGGCTGAGGAGTGTG---GGCGCACGGCGCAGGA-------------C 580
CT49_LTR12E        CCGCCCCCTGCTCCGTGGCACCCGGTCCCATCTACCACCCAAGAGCTGAGGAGTGCA---GGTGCGTGGTGTGGGA-------------C 570
PTPN13_LTR12EC     CCGCCCCCTGCTCCACAGCACCCAGTCCCATTGACCACCCAAGGGCTGAGGAATGCC------------GGCGGCA-------------- 507
RADIL_LTR12C       TTGCCACCTGCTC-AGGGCACCCAGTCCCATCGACCACCCAAGGGCTGAGGAGTGCCTTTGGAGCAGGGCACGGGA-------------C 542
Clustal Consens                                                                                               1

ADH1C_LTR12        --------------------------------------TGAGAGGTGAA-GCCAGCTGGACTTCTGGGTCAGGTAGGGACTTGGAGAA-- 49
GBP5_LTR12C        TGGCAGGCAGCTCCACCTGCCGCCCG--GTGCAAGATCCACTGGGTGAA-GCCAGCTGGGCTCTTGAGTCTAGTGGGAACTTGGAGAA-- 630
SEMA4D_LTR12C      TGGCAGGCAGCTCCACCTGCAGCCCC-GGTGCGGGATCCACTAGGTGAA-ACCAGCTGGGCTCCTGAGTCTGGTGGGGACGTGGAGAG-- 670
TNFRSF10B_LTR12    TGGCAGGCAGCTCCACCTGCAACCCC-GGTGCGGGATCCACTGGGTGAC-GACACCTGGGCTCCTGAGTCTGGTGGGGACGTGGAGAA-- 678
TP63_LTR12C        TGGCAGGCAGCTCCATCTGCAGCCCC-GGTGGGAGATCCACTGGGCGAA-GCCAGCTGGCCTCCTGAGT--------------------- 649
DHRS2_LTR12D_1     ------------------------------------------AGGTGAA-GCCGGCTGGGCTTCTGGGTCCGGTGGGGACCTGGAGAA-- 383
DHRS2_LTR12D_2     -------------------------------------------TCTATG-TCTAGCTAA---------TCAGGTGGGGACTTGGAGAA-- 35
NR1H4_LTR12C       TGGCAGGCAGCTCCACCTGCAGCCCC-GGTGCGGGATCCACTGGGTGAA-GCCAGCTGGGCTCCTGAGTAGGGTGGGGACGTGGAGAA-- 669
C9orf53_LTR12CD    TGGCAGGCAGCTCCACCTGCAGCCCT-GGTGCAGGATCCACTAGGTGAA-GCCAGCTGGGCTCCTGAGT--------------------- 642
CPED1_LTR12C       TGGCAGGCAGCTCCACCTGCAGCCCCCGGTGCGGGATCCACTGGGTGAA-GCCAGCTGAGCTCCTGAGTCTGGTGGGGACGTGGAGAA-- 635
C9orf85_LTR12C     TGGCAGGCAGCTCCATCTGCAGCCCC-GGAGTGGGATCCACGGGGTGAA-GCCAGCTGGGCTCCTGAGTCTGGTGGGGACGTGGAGAA-- 667
CCR4_LTR12C        TGGCAGACAGCTCCACCTGCAGCCCC-AGTGCGGGATCCACTGGGTGAA-GACAGCTGGGCTCCTGAGTCTGGTAGGGATGTGGAG---- 666
ACSBG1_LTR12C      TGGCAGGCAGCTCCACCTGCAGCCCG-GGTGCGGGATCCACTGGGTGAA-CTCAGCTGGGCTCCTAAGTCTGGTGGGGCCTTGGAGAA-- 670
KCNN3_LTR12C       TGGCAGGCAGCTCCACCTGCAGCCCT-GATGTGGGATCCACTGGGTGAA-GCCAGCTGGGCTCCTGAGTCTGGTGGGGACGTGGAGAA-- 680
CSF3_LTR12CD       TGGCAGGCAGCTACCCCTGCAGCCCT-GGTGCGGAATCCACTGGGTGAA-GCCAGCTGGGCTCCTGAGTCTGGTGGAGACTTGGAGAACC 672
TMOD1_LTR12EB      TGGCAGGCAGCTCCACCTGCGGCCCC-GGTGCAGGATCCACTGGGTGAA-GCCAGCTGGGCTCCTGAGTCTGGTGGGGACTTGGAGAA-- 624
SLC36A2_LTR12      --------------------------------------TGAGAGGTGAA-GCCGGCTGGGCTTCTGGGTCGGGTGGGGACTTGGAGAA-- 49
IER3_LTR12C        TGGCAGGCAGCTCCACCTGCAGCCCC-AGTGTGGGGTCCACTAGGTGAA-GCCAGCTGGGCTCCTGAGTCTGGTGAGGATGTGGAGAG-- 670
PIK3C2G_LTR12C     TGGCAGGCAGCTCCACCTGCAGCCCC-AATGCGAGATCCACTGGGTGAC-GCCAGCTGGGCTCCTGAGT--------------------- 647
CT49_LTR12E        TGGCGGGCCGCTCTGCCCACAGCCCT-GGCACAGGATCCACTAGGCGAA-GCCAGCTGGCGTCCTGAGTTGGGTGGGGACTTGGAGAA-- 656
PTPN13_LTR12EC     -GGCAGGGAGCTCCACCTGCAGCCTC-CATTCAGGATCCACTGGGTGAA-GCCAGCTGGGCTCCTGAGTCTAGTGGGGATGTGGAGAA-- 592
RADIL_LTR12C       TGGCAGGCAGCTCTACCTGCAGCCCC-TGTGCAGGATCCACTGGGTGAAAGCCAGCTGGGCTCCTGAGTCTGGTGGGGACTTGGAGAA-- 629
Clustal Consens                                                           **           *                      3
```

```
ADH1C_LTR12         ------------------------------------------------------------------------------------------ 49
GBP5_LTR12C         ------------------------------------------------------------------------------------------ 630
SEMA4D_LTR12C       ------------------------------------------------------------------------------------------ 670
TNFRSF10B_LTR12     ------------------------------------------------------------------------------------------ 678
TP63_LTR12C         ------------------------------------------------------------------------------------------ 649
DHRS2_LTR12D_1      ------------------------------------------------------------------------------------------ 383
DHRS2_LTR12D_2      ------------------------------------------------------------------------------------------ 35
NR1H4_LTR12C        ------------------------------------------------------------------------------------------ 669
C9orf53_LTR12CD     ------------------------------------------------------------------------------------------ 642
CPED1_LTR12C        ------------------------------------------------------------------------------------------ 635
C9orf85_LTR12C      ------------------------------------------------------------------------------------------ 667
CCR4_LTR12C         ------------------------------------------------------------------------------------------ 666
ACSBG1_LTR12C       ------------------------------------------------------------------------------------------ 670
KCNN3_LTR12C        ------------------------------------------------------------------------------------------ 680
CSF3_LTR12CD        TTTATGTCTAGCTCAGGGATCGTAAATACACCAATCAGCACCCTGTGTCTAGCTCAGGTTCTGTGAATGCACCAATCCACACTCTGTATC 762
TMOD1_LTR12EB       ------------------------------------------------------------------------------------------ 624
SLC36A2_LTR12       ------------------------------------------------------------------------------------------ 49
IER3_LTR12C         ------------------------------------------------------------------------------------------ 670
PIK3C2G_LTR12C      ------------------------------------------------------------------------------------------ 647
CT49_LTR12E         ------------------------------------------------------------------------------------------ 656
PTPN13_LTR12EC      ------------------------------------------------------------------------------------------ 592
RADIL_LTR12C        ------------------------------------------------------------------------------------------ 629
Clustal Consens                                                                                                3

ADH1C_LTR12         ---------------------------CTTTTCTGTCTAGCTAAAGGATTGTAAACACACCAATCAGTGCTCTGT--------------- 97
GBP5_LTR12C         ---------------------------TCTTTATGTCTAGCTAAGGGATTGTAAATACACCAATCAGCACTCTGTATCTATTTAATCTGG 693
SEMA4D_LTR12C       ---------------------------TCTTTATATCTAGCTCAGGGATTGTAAATACACCAATCAGCACCCTGT--------------- 718
TNFRSF10B_LTR12     ---------------------------CCTTTATGTTTAGCTAAGGGATTGTAAATACACCAATTGGCACTGTGT--------------- 726
TP63_LTR12C         ------------------------------------------------------------------------------------------ 649
DHRS2_LTR12D_1      ---------------------------CTTTTCTGTCTAGCTAAAGGTTTGTAAATGCACCCATCAGCACTCTGT--------------- 431
DHRS2_LTR12D_2      ---------------------------CTTTTGTGTCTAGCGAAAGGATTGTAAATGCACCAATCAGCACTCTGT--------------- 83
NR1H4_LTR12C        ---------------------------CCTTTATGTCTAGCTCAGGGATTGTAAATACACCAATTGGCACTCTGT--------------- 717
C9orf53_LTR12CD     ------------------------------------------------------------------------------------------ 642
CPED1_LTR12C        ---------------------------TCTTCATGTCTAGCTCAGGGATTGTAAATACACCAATCGGCACTCTGT--------------- 683
C9orf85_LTR12C      ---------------------------CCTTTATGTCTAGCTCAGGGATTGTAAATACACCAATCAGCACCCTGT--------------- 715
CCR4_LTR12C         ---------------------------TCTTTATATCTAGCTCAGGGATTGTAAGCACACCAATCAGCACCCTGT--------------- 714
ACSBG1_LTR12C       ---------------------------CCTTTATGTCTAGCTCAGGGATTGTAAGTACACCAATCAGCACTCTGT--------------- 718
KCNN3_LTR12C        ---------------------------CCTTTATGTCTAGCCCAGGGATTGTAAATACACCAATCGGCACTCTGT--------------- 728
CSF3_LTR12CD        TAGCTACTCTGATGGGGCCTTGGAGAACCTTTATGTCTAGCTCAGGGATTGTAAATACACCAATCGGCACTCTGT--------------- 837
TMOD1_LTR12EB       ---------------------------TCTTTATGTCTGGCTAAGGGATGGCAAATCCACCAATCAGCACTCTGT--------------- 672
SLC36A2_LTR12       ---------------------------CTTTTCTGTCTAGCTAAAAGATTGTAAATGTACCAATCAGCGCTCTGT--------------- 97
IER3_LTR12C         ---------------------------TCTTTATGTCTAGCTCAGGGATTGTAAATACACCAATCAGCACCCTGT--------------- 718
PIK3C2G_LTR12C      ------------------------------------------------------------------------------------------ 647
CT49_LTR12E         ---------------------------CTTTTATGTCTAGGTAGAGGATTGTAAATGCACCAATCAGCACTCTGT--------------- 704
PTPN13_LTR12EC      ---------------------------CCTTTATATCTAGCTAAGGGATTGTAAATACACCAATCAGCACTCTGT--------------- 640
RADIL_LTR12C        ---------------------------CCTTTATGTCTAGCTAAGGGATTGTAAATAAACCAATTGGCACTCTGT--------------- 677
Clustal Consens                                                                                                3

ADH1C_LTR12         ----------------------GTCTAGCTAAAAGTTTGTAAACACAACAATCAGCACTCTGTAA----------------AAATGCACT 149
GBP5_LTR12C         TGGGGACTTGGAGAATCTTTATGTCTAGCTAAGGGATTGTAAATGCACCAATCAGCACTCTGTATCTACCTCAAGGTTTGTAAATGCACC 783
SEMA4D_LTR12C       ----------------------GTTTAGCTCAAGGTTTGTGAGTGCACCAATCGACAC-------------------------------- 754
TNFRSF10B_LTR12     ----------------------ATCTAGCTCAAGTTTTGTAAACACACCAATCAGCACCGTGTGTCTAGCTCAGGGTTTGTGAATGCACC 794
TP63_LTR12C         ------------------------------------------------------------------------------------------ 649
DHRS2_LTR12D_1      ----------------------GTCTAGCTAAAGGTTTGTAAATGCACCAATCAGCAA-------------------------------- 467
DHRS2_LTR12D_2      ----------------------GTCTAGCTAAAGGTTTGTAAACACAGCAATCAGCACTCTGTCA----------------AAATGGACC 135
NR1H4_LTR12C        ----------------------GTCTAGCTCAAGGTTTGTAAACACACCAATCAGCACCCTGTGTCTAGCTCAGGGTTTGTGAACGCACC 785
C9orf53_LTR12CD     ------------------------------------------------------------------------------------------ 642
CPED1_LTR12C        ----------------------ATCTAGCTCAAGGTTTGTAAACACACCAATCAGCACCCTGTGTCTAGCTCAGGGTTTGTGAATGCACC 751
C9orf85_LTR12C      ----------------------GTCTAGCTCAGGGTTTGTGAATGCACCAATCGACAC-------------------------------- 751
CCR4_LTR12C         ----------------------GTTTAGCTCAAGGTTTGTGAATGCACCAATGGACAC-------------------------------- 750
ACSBG1_LTR12C       ----------------------ATCTAGCTCAAGGTTTGTAAACACACCAATCAGCACCCTGTGTCTAGCTCAGGGTTTGTGAATGCACC 786
KCNN3_LTR12C        ----------------------ATCTAGCTCAAGGTTTGTAAACACACCAATCAGCACCCTGTGTCTAGCTCAGGGTTTGTGAATGCACC 796
CSF3_LTR12CD        ----------------------ATCTAGCTCAAGGTTTGTAAACACACCAATCAGCACCCTGTGTCTAGCTCAGGGTATGTGAATGCACC 905
TMOD1_LTR12EB       ----------------------ATCTAGCTCAAGGTTTGTAAACACACCAATCAGCACCCTGTGTCTAGCTCAGGGTTTGCAAATGCACC 740
SLC36A2_LTR12       ----------------------GTCTAGCTAAAGGTTTGTAAGCACACCAATCAGCACTCTGTAA----------------ATATGCACC 149
IER3_LTR12C         ----------------------GTTTAGCTCAAGGCTTGTGAGTGCACCAGTCCACAC-------------------------------- 754
PIK3C2G_LTR12C      ------------------------------------------------------------------------------------------ 647
CT49_LTR12E         ----------------------GTCTAGCTCAAGGTTTGTAAATGCACCAATCAGTGC-------------------------------- 740
PTPN13_LTR12EC      ----------------------ATCTAGCTCAAGGTTTGTAAACACACCAATCAGCAC-------------------------------- 676
RADIL_LTR12C        ----------------------ATCTAGCTCAAGGTTTGTAAACACACCAATCAGCACCTTGTGTCTAGCTCAGGGTTTGTGAATGCACC 745
Clustal Consens                                                                                                3

ADH1C_LTR12         AATCAGTGCTCTGTGTCTAGCTAAAGGTTTG----------------------------------------------------------- 180
GBP5_LTR12C         AATCAGCACTCTGTATCTAGCTCAAGGTTTG----------------------------------------------------------- 814
SEMA4D_LTR12C       ---------TCTGTATCTAGCTGCTCTGGTGAGGACGTGGAGAACCTTTATATCTAGCTCAGGGATTGTAAATACACCAATCAGCACCCT 835
TNFRSF10B_LTR12     AATTGACACTCTGTATCTAGCTAGTCTGGTG----------------------------------------------------------- 825
TP63_LTR12C         ------------------------------------------------------------------------------------------ 649
DHRS2_LTR12D_1      ---------TCTGTGTCTAGCCAATCTGGTG----------------------------------------------------------- 489
DHRS2_LTR12D_2      AATCAGCTCTCTATA--------------------------------------------------------------------------- 150
NR1H4_LTR12C        AATTGACA----CTCTGTAGCTACTCTGGTG----------------------------------------------------------- 812
C9orf53_LTR12CD     ------------------------------------------------------------------------------------------ 642
CPED1_LTR12C        AGTCCAC--TCTGTATCTAGCTATTCTGGAG----------------------------------------------------------- 780
C9orf85_LTR12C      -----------TGTATCTAGCTACTCTGGTG----------------------------------------------------------- 771
CCR4_LTR12C         ---------TCTGTATCTAGCTGCCCTGGTG----------------------------------------------------------- 772
ACSBG1_LTR12C       AATCGACACTCTGTATCTAGCTACTCTGGTG----------------------------------------------------------- 817
KCNN3_LTR12C        AATCCACAATCTGTATCTAACTACTCTGGTG----------------------------------------------------------- 827
CSF3_LTR12CD        AATCGACAGTCTGTATCTGGCTACTTTCATGGGCATCCGTGTGAAGAGACCACCAAACAGGCTTTGTGTGAGCAATAAAGCTTTTATCAC 995
TMOD1_LTR12EB       AATTGACA-CCTGTATCTAGCTACTCTGGTG----------------------------------------------------------- 770
SLC36A2_LTR12       AATCAGTGCTCTGTGTCTAGCTAATCAGGTG----------------------------------------------------------- 180
IER3_LTR12C         ---------TCTGTATCTAGTTGCTCTGGTG----------------------------------------------------------- 776
PIK3C2G_LTR12C      ------------------------------------------------------------------------------------------ 647
CT49_LTR12E         ---------TCTGTGTCTAGCTAATCTAGTG----------------------------------------------------------- 762
PTPN13_LTR12EC      ---------CCTGTGTCTAGCTC------------------------------------------------------------------- 690
RADIL_LTR12C        AATAGACACTCTGTATCTAGCTACTCTGGTG----------------------------------------------------------- 776
Clustal Consens                                                                                                3
```

```
ADH1C_LTR12       ------------------------------------------------------------------------------------------ 180
GBP5_LTR12C       ------------------------------------------------------------------------------------------ 814
SEMA4D_LTR12C     GTGTTTAGCTCAAGGTTTG----------------------------------------------------------------------- 854
TNFRSF10B_LTR12   ------------------------------------------------------------------------------------------ 825
TP63_LTR12C       ------------------------------------------------------------------------------------------ 649
DHRS2_LTR12D_1    ------------------------------------------------------------------------------------------ 489
DHRS2_LTR12D_2    ------------------------------------------------------------------------------------------ 150
NR1H4_LTR12C      ------------------------------------------------------------------------------------------ 812
C9orf53_LTR12CD   ------------------------------------------------------------------------------------------ 642
CPED1_LTR12C      ------------------------------------------------------------------------------------------ 780
C9orf85_LTR12C    ------------------------------------------------------------------------------------------ 771
CCR4_LTR12C       ------------------------------------------------------------------------------------------ 772
ACSBG1_LTR12C     ------------------------------------------------------------------------------------------ 817
KCNN3_LTR12C      ------------------------------------------------------------------------------------------ 827
CSF3_LTR12CD      CTGGGTGCAGGTGGGCTGAGTCCGAAAAGAGAGTCAGCGAAGGGAGATAAGGGTGGGGCCGTTTTATAGGATTTGGGTAGGTAAAGGAAA 1085
TMOD1_LTR12EB     ------------------------------------------------------------------------------------------ 770
SLC36A2_LTR12     ------------------------------------------------------------------------------------------ 180
IER3_LTR12C       ------------------------------------------------------------------------------------------ 776
PIK3C2G_LTR12C    ------------------------------------------------------------------------------------------ 647
CT49_LTR12E       ------------------------------------------------------------------------------------------ 762
PTPN13_LTR12EC    ------------------------------------------------------------------------------------------ 690
RADIL_LTR12C      ------------------------------------------------------------------------------------------ 776
Clustal Consens                                                                                              3

ADH1C_LTR12       ------------------------------------------------------------------------------------------ 180
GBP5_LTR12C       ------------------------------------------------------------------------------------------ 814
SEMA4D_LTR12C     ------------------------------------------------------------------------------------------ 854
TNFRSF10B_LTR12   ------------------------------------------------------------------------------------------ 825
TP63_LTR12C       ------------------------------------------------------------------------------------------ 649
DHRS2_LTR12D_1    ------------------------------------------------------------------------------------------ 489
DHRS2_LTR12D_2    ------------------------------------------------------------------------------------------ 150
NR1H4_LTR12C      ------------------------------------------------------------------------------------------ 812
C9orf53_LTR12CD   ------------------------------------------------------------------------------------------ 642
CPED1_LTR12C      ------------------------------------------------------------------------------------------ 780
C9orf85_LTR12C    ------------------------------------------------------------------------------------------ 771
CCR4_LTR12C       ------------------------------------------------------------------------------------------ 772
ACSBG1_LTR12C     ------------------------------------------------------------------------------------------ 817
KCNN3_LTR12C      ------------------------------------------------------------------------------------------ 827
CSF3_LTR12CD      ATTACAGTCAAAGGGGGTTTGTTCTCTGGCGGGCAGGAGTGGGGGGTCGCAAGGTGCTCAGTGGGGGTGCTTTTTGAGCCAGGATGAGCC 1175
TMOD1_LTR12EB     ------------------------------------------------------------------------------------------ 770
SLC36A2_LTR12     ------------------------------------------------------------------------------------------ 180
IER3_LTR12C       ------------------------------------------------------------------------------------------ 776
PIK3C2G_LTR12C    ------------------------------------------------------------------------------------------ 647
CT49_LTR12E       ------------------------------------------------------------------------------------------ 762
PTPN13_LTR12EC    ------------------------------------------------------------------------------------------ 690
RADIL_LTR12C      ------------------------------------------------------------------------------------------ 776
Clustal Consens                                                                                              3

ADH1C_LTR12       ------------------------------------------------------------------------------------------ 180
GBP5_LTR12C       ------------------------------------------------------------------------------------------ 814
SEMA4D_LTR12C     ------------------------------------------------------------------------------------------ 854
TNFRSF10B_LTR12   ------------------------------------------------------------------------------------------ 825
TP63_LTR12C       ------------------------------------------------------------------------------------------ 649
DHRS2_LTR12D_1    ------------------------------------------------------------------------------------------ 489
DHRS2_LTR12D_2    ------------------------------------------------------------------------------------------ 150
NR1H4_LTR12C      ------------------------------------------------------------------------------------------ 812
C9orf53_LTR12CD   ------------------------------------------------------------------------------------------ 642
CPED1_LTR12C      ------------------------------------------------------------------------------------------ 780
C9orf85_LTR12C    ------------------------------------------------------------------------------------------ 771
CCR4_LTR12C       ------------------------------------------------------------------------------------------ 772
ACSBG1_LTR12C     ------------------------------------------------------------------------------------------ 817
KCNN3_LTR12C      ------------------------------------------------------------------------------------------ 827
CSF3_LTR12CD      AGGAAAAGGACTTTCACAAGGTAATGTCATCAATTAAGGCAAGGACCCGCCATTTACACCTCTTTTGTGGTGGAATGTCATCAGTTAAGT 1265
TMOD1_LTR12EB     ------------------------------------------------------------------------------------------ 770
SLC36A2_LTR12     ------------------------------------------------------------------------------------------ 180
IER3_LTR12C       ------------------------------------------------------------------------------------------ 776
PIK3C2G_LTR12C    ------------------------------------------------------------------------------------------ 647
CT49_LTR12E       ------------------------------------------------------------------------------------------ 762
PTPN13_LTR12EC    ------------------------------------------------------------------------------------------ 690
RADIL_LTR12C      ------------------------------------------------------------------------------------------ 776
Clustal Consens                                                                                              3

ADH1C_LTR12       ------------------------------------------------------------------------------------------ 180
GBP5_LTR12C       ------------------------------------------------------------------------------------------ 814
SEMA4D_LTR12C     --------------------------------------------------------------------------------------TGAG 858
TNFRSF10B_LTR12   ------------------------------------------------------------------------------------------ 825
TP63_LTR12C       ------------------------------------------------------------------------------------------ 649
DHRS2_LTR12D_1    ------------------------------------------------------------------------------------------ 489
DHRS2_LTR12D_2    ------------------------------------------------------------------------------------------ 150
NR1H4_LTR12C      ------------------------------------------------------------------------------------------ 812
C9orf53_LTR12CD   ------------------------------------------------------------------------------------------ 642
CPED1_LTR12C      ------------------------------------------------------------------------------------------ 780
C9orf85_LTR12C    ------------------------------------------------------------------------------------------ 771
CCR4_LTR12C       ------------------------------------------------------------------------------------------ 772
ACSBG1_LTR12C     ------------------------------------------------------------------------------------------ 817
KCNN3_LTR12C      ------------------------------------------------------------------------------------------ 827
CSF3_LTR12CD      TGGGGCAGGGCATATTCACTTCTTTTGTGATTCTTCAGTTACTTCAGGCCATCTGGGCGTATATGTGCAAGTTACAGGGGATGCGATGGC 1355
TMOD1_LTR12EB     ------------------------------------------------------------------------------------------ 770
SLC36A2_LTR12     ------------------------------------------------------------------------------------------ 180
IER3_LTR12C       ------------------------------------------------------------------------------------------ 776
PIK3C2G_LTR12C    ------------------------------------------------------------------------------------------ 647
CT49_LTR12E       ------------------------------------------------------------------------------------------ 762
PTPN13_LTR12EC    ------------------------------------------------------------------------------------------ 690
RADIL_LTR12C      ------------------------------------------------------------------------------------------ 776
Clustal Consens                                                                                              3
```

```
ADH1C_LTR12        ----------------------------------------------TAAACGCACCAATCAGCACTCTGTAA------------AAATGC 212
GBP5_LTR12C        ----------------------------------------------TAAATAGGCCAATCCACACTCTGTATCTAGCTAATCTAGTGGGG 858
SEMA4D_LTR12C      TGCACCAATCGACACTCTGTATCTAGCTGCTCTGGTGGGGCCTTGGAGAACCTGTGTGTGGAAACTCTGTATCTAACTAATCTGATGGGG 948
TNFRSF10B_LTR12    -------------------------------------GGGCCTTGGAGAACCTTTGTGTCCACACTCTGTATCTAGCTAATCTGGTGGGG 878
TP63_LTR12C        ---------------------------------------------------------------------------------CTGGTGGGG 658
DHRS2_LTR12D_1     -------------------------------------GGGACTTGGAGAACTTTTGTGTC---------TAGCTAAAGGATTGTAAATGC 533
DHRS2_LTR12D_2     ------------------------------------------------------------------------------------------ 150
NR1H4_LTR12C       -------------------------------------GGGACTTGGAGAACCTTTGTGTGGACACTCTGTATCTAGCTAATCTGGTGGGG 865
C9orf53_LTR12CD    ---------------------------------------------------------------------------------CTGGTGGGG 651
CPED1_LTR12C       -------------------------------------GGGCCTTGGAGAACCTTTGTGTGGACACTCTGTATCTAGCTAATCTGGTGGGG 833
C9orf85_LTR12C     -------------------------------------GGGCCTTGGAGAACCTTTGTATAGACACTCTGTATCTAGCTAATCTGGTGGGG 824
CCR4_LTR12C        -------------------------------------GGGACGTGGAGAACCTTTATGTC---------TAGCTCAGGGATTGTAAATAC 816
ACSBG1_LTR12C      -------------------------------------GGGCCTTGGAGAACCTTTATGTCCACACTCTGTATCTAGCTAATCTGGTGGGG 870
KCNN3_LTR12C       -------------------------------------GGGACTTGGAGAACCTTTGTGTGGACACTCTGTATCTAGCCCATCTAGTGAGG 880
CSF3_LTR12CD       TTGGCTTGGGCTCAGAGGCTTGACAGCTACTCTGGTGGGGCCTTGGAGAATGTTTGTGTCGACACTCTGTATCTAGTTAATCTAGTGGGG 1445
TMOD1_LTR12EB      -------------------------------------GGGACATGGAGAACTTTTGTGTCCACACTCTGTATCTAGCTAATCTAGTGGGG 823
SLC36A2_LTR12      ----------------------------------------------GGGACTTGGAGAACTTTTCTGTCTAGCTAAAGGATTGTAAATGT 224
IER3_LTR12C        -------------------------------------GAGCCTTGGAGAACCTGTGTGTCCAAACTCTGTATCTAACTAATCTGATGGGG 829
PIK3C2G_LTR12C     ---------------------------------------------------------------------------------CTGGTAGGG 656
CT49_LTR12E        -------------------------------------GGGACTTGGAGAACTTTTGTGTC---------TAGCTAAAGGATTGTAAATGC 806
PTPN13_LTR12EC     --------------------------------------AGGGTTTGTGAATGCACCAATCGACACTCTGTATTTAGCTACTCTGGTGGGG 742
RADIL_LTR12C       -------------------------------------GGGACTTGGAAAACCTTTTTGTCAACACTCTGTATCTAGTTAATCTGGTGGGG 829
Clustal Consens                                                                                               3

ADH1C_LTR12        ACCAATCAGTGCTCTGTGTCTAGCTAAAGGTTTGTAAAT-----------------GCACCAATCAGCACTCTGTAAAAACGGACCAATC 285
GBP5_LTR12C        ACATGAAGAACTTTTGTGTCCAGCTCAGGGATTGTAAAC-----------------ACACCAATCAGCACCCTGTCAAAACAGACCAATC 931
SEMA4D_LTR12C      ACGTGGAGAACCTTTCTATCTAGCTCAGGGATTGTAAAC-----------------GCACCAATCAGCGCCCTGACA------------- 1008
TNFRSF10B_LTR12    AAGTGGAGAACATTTGTGTCTAGCTCAGGGATTGTAAA------------------CCACCAATCAGCGCCCTGTCA------------- 937
TP63_LTR12C        ACGTGGAGAATCTTTATGTCTAGCTCAGGGATTGTAAAT-----------------ACACCAATCAGTGCCCTGTCA------------- 718
DHRS2_LTR12D_1     ACCAATCAGCACTCTGTGTCTAGCTAAAGGTTTGTAAAT-----------------GCACCAATCAGCACTCTGTCA------------- 593
DHRS2_LTR12D_2     ------------------------------------------------------------------------------------------ 150
NR1H4_LTR12C       AAGTGGAGAACCTTTGTGTCTAGCTCAGGGATTGTAAAG-----------------GCACCAATCAGCGCCCTGTCA------------- 925
C9orf53_LTR12CD    ACGTGGAGAACCTTTGTATCTAGTTCAGGGATTGTAAAC-----------------GCACCAATCAGCGCCCTGACA------------- 711
CPED1_LTR12C       AGGTGGAGAACCTTTGTGTCTAGCTCAGGGATTGTAAAC-----------------GCACCAATCAGTGCCCTGTCA------------- 893
C9orf85_LTR12C     ACGTGGAGAACCTTTGTGTCTAGCTTAGGGATTGTAAACGCA-----------------CCAGTCAGCGCCCTGTCA------------- 884
CCR4_LTR12C        ACCAATCAGCACCCTGTGTTTAGCTCAAGGTTTGTGAGT-----------------GCACCAATCGACACTCTGTATCTGGCTGCTCTGG 889
ACSBG1_LTR12C      ATGTGGAGAACCTTTGTGTCTAGCTCAGGGATTGTAAAC-----------------GCACCAATCAGCGCCCTGTCA------------- 930
KCNN3_LTR12C       AGGTGGAGAACCTTTGAGTCTAGCTCTGGGATTGTAAACGCATTGGGATTGTAAATGCACCAATCAGTGCCCTGTCA------------- 957
CSF3_LTR12CD       ACGTGGAGAACCTTTGTGTCTAGCTCAGGGATTGTAAAC-----------------GCACCAATCAGCGCCCTGTCA------------- 1505
TMOD1_LTR12EB      AGGTGGAGAACTTTTGTGTCTAGCTCAGGGATTGTAAACGCA-----------------CCAGTCAGCACCCTGTCA------------- 883
SLC36A2_LTR12      ACCAATCAGTGCTCTGTGTCTAGCTAAAGGTTTGTAAAC-----------------GCACCAATCAGCACTCTGTAAAAACGCACCAATC 297
IER3_LTR12C        ACGTGGAGAACCTTTGTATCTAGCGCAAGGATTGTAAAC-----------------GCACCAATCAGCACCCTGACA------------- 889
PIK3C2G_LTR12C     ACGTG---AACCTTTACCTCTACCTCAGGGATTGTAAAT-----------------GCACCAATCAGCGCCGTGTCA------------- 713
CT49_LTR12E        ACCAATCAGCACTCTGTGTCTAGCTCAAGGTTTGTAAAT-----------------GCACCAATCAGCACCCTGTCA------------- 866
PTPN13_LTR12EC     CCTTAGAGAACCTTTATGTCTAGCTCAGGGATTGTAAAC-----------------GCACCAATCAGCGCCCTGTCA------------- 802
RADIL_LTR12C       ACATGGAGAACCTTTGTGTCTAGCTCAGGGATTGTAAAC-----------------GCACCAATCAGCACCCTGTCA------------- 889
Clustal Consens                                                                                               3

ADH1C_LTR12        AGCACTCTGTA----------------------------------------------------------------------AAATGGACC 305
GBP5_LTR12C        AGCTCTCTGTA----------------------------------------------------------------------AAATGGACC 951
SEMA4D_LTR12C      ---------------------------------------------------------------------------------AAACAGGCC 1017
TNFRSF10B_LTR12    ---------------------------------------------------------------------------------AAACAGACC 946
TP63_LTR12C        ---------------------------------------------------------------------------------AAACAGACC 727
DHRS2_LTR12D_1     ---------------------------------------------------------------------------------AAACGGACC 602
DHRS2_LTR12D_2     ---------------------------------------------------------------------------------AAACAGACC 159
NR1H4_LTR12C       ---------------------------------------------------------------------------------AAACAGACC 934
C9orf53_LTR12CD    ---------------------------------------------------------------------------------AAACAGGCC 720
CPED1_LTR12C       ---------------------------------------------------------------------------------AAACAGACT 902
C9orf85_LTR12C     ---------------------------------------------------------------------------------ACACAGACC 893
CCR4_LTR12C        TGGGGAGGTGGAGAACCTTTATGTCTAGCTCAGGGATTGTAAATACACCAATCGGCACTGTGTATCCAGCTCAAGGTTTGTAAACACACC 979
ACSBG1_LTR12C      ---------------------------------------------------------------------------------AAACAGACC 939
KCNN3_LTR12C       ---------------------------------------------------------------------------------AAACAGACC 966
CSF3_LTR12CD       ---------------------------------------------------------------------------------AAACAGACC 1514
TMOD1_LTR12EB      ---------------------------------------------------------------------------------AAACAGACC 892
SLC36A2_LTR12      AGCGCTCTGTGTTTA-----------------------------------------------------GCTAAAGGTTTGTAAACGCACC 334
IER3_LTR12C        ---------------------------------------------------------------------------------AAACAGGCC 898
PIK3C2G_LTR12C     ---------------------------------------------------------------------------------AAACAGACC 722
CT49_LTR12E        ---------------------------------------------------------------------------------AAACGGACC 875
PTPN13_LTR12EC     ---------------------------------------------------------------------------------AAACAGACC 811
RADIL_LTR12C       ---------------------------------------------------------------------------------AAACAGACC 898
Clustal Consens                                                                                     * *    *  6

ADH1C_LTR12        AATCAGCGCTCTGTAAAA------------------------------------------------------------------------ 323
GBP5_LTR12C        AATCGGCTCTCTGTAAAA------------------------------------------------------------------------ 969
SEMA4D_LTR12C      ACTCGGCT---------------------------------------------------------------------------------- 1027
TNFRSF10B_LTR12    ACTCCG------------------------------------------------------------------------------------ 952
TP63_LTR12C        ACTCGG------------------------------------------------------------------------------------ 733
DHRS2_LTR12D_1     AATCAGCTCTCTGTA-----------------AAACAGACCAATCAGCTCTCTGTA---------------------------------- 641
DHRS2_LTR12D_2     AATCAGCTCTCTGTAAAA------------------------------------------------------------------------ 177
NR1H4_LTR12C       ACTGGG------------------------------------------------------------------------------------ 940
C9orf53_LTR12CD    ACTGGG------------------------------------------------------------------------------------ 726
CPED1_LTR12C       ACTGGGCTCTACCAG--------------------------------------------------------------------------- 917
C9orf85_LTR12C     ACTGGG------------------------------------------------------------------------------------ 899
CCR4_LTR12C        AATCAGCACCCTGTGTTTAGCTCAAGGTTTGTGAATGCACCAATCGACACTCTGTATCTAGCTGCTCTGGTGGGGCCTTGGAGAACCTGT 1069
ACSBG1_LTR12C      ACTGGG------------------------------------------------------------------------------------ 945
KCNN3_LTR12C       ACTCGG------------------------------------------------------------------------------------ 972
CSF3_LTR12CD       ACTCGG------------------------------------------------------------------------------------ 1520
TMOD1_LTR12EB      AACCAGCTCTCTATA--------------------------------------------------------------------------- 907
SLC36A2_LTR12      AATCAGCACTCTGTAAAAA----------------------------------------------------------------------- 353
IER3_LTR12C        ACTCGGCT---------------------------------------------------------------------------------- 908
PIK3C2G_LTR12C     ACTCGG------------------------------------------------------------------------------------ 728
CT49_LTR12E        AATCAGCTCTTGGTA-----------------AAATGGACCAATCAGCTCTCTGTA---------------------------------- 914
PTPN13_LTR12EC     ACTCGG------------------------------------------------------------------------------------ 817
RADIL_LTR12C       ACTCGG------------------------------------------------------------------------------------ 904
Clustal Consens    *    *                                                                                     8
```

```
ADH1C_LTR12       ------------------------------------------------------------------------------------------ 323
GBP5_LTR12C       ------------------------------------------------------------------------------------------ 969
SEMA4D_LTR12C     ------------------------------------------------------------------------------------------ 1027
TNFRSF10B_LTR12   ------------------------------------------------------------------------------------------ 952
TP63_LTR12C       ------------------------------------------------------------------------------------------ 733
DHRS2_LTR12D_1    ----------------------------------------------------------------------AAATGGACCAATCAGCTCTC 661
DHRS2_LTR12D_2    ------------------------------------------------------------------------------------------ 177
NR1H4_LTR12C      ------------------------------------------------------------------------------------------ 940
C9orf53_LTR12CD   ------------------------------------------------------------------------------------------ 726
CPED1_LTR12C      ------------------------------------------------------------------------------------------ 917
C9orf85_LTR12C    ------------------------------------------------------------------------------------------ 899
CCR4_LTR12C       GTGTCCAAATTCTGTATCTAACTAATCTGATGGGACGTGGAGAACGTTTGTATCTAGCTCAGGGATTGTAAACGCACCAATCAGCGCCC 1159
ACSBG1_LTR12C     ------------------------------------------------------------------------------------------ 945
KCNN3_LTR12C      ------------------------------------------------------------------------------------------ 972
CSF3_LTR12CD      ------------------------------------------------------------------------------------------ 1520
TMOD1_LTR12EB     ------------------------------------------------------------------------------------AAATGG 913
SLC36A2_LTR12     ------------------------------------------------------------------------------------------ 353
IER3_LTR12C       ------------------------------------------------------------------------------------------ 908
PIK3C2G_LTR12C    ------------------------------------------------------------------------------------------ 728
CT49_LTR12E       ----------------------------------------------------------------------AAATGGACCAATTAGATCTC 934
PTPN13_LTR12EC    ------------------------------------------------------------------------------------------ 817
RADIL_LTR12C      ------------------------------------------------------------------------------------------ 904
Clustal Consens                                                                                              8
                                                                               TATA1
ADH1C_LTR12       ---------------------TGGACCAATCAGCAGGACATGGGGTGGGGGGGGCCAAATAAGGGAATAAAAGCTGGCCACCCAAGACAG 392
GBP5_LTR12C       ---------------------TGGACCCATCAGCAGGAT-----GTGGGTGGGGCCAGATAAGAGAA---------------GTAGCCAG 1018
SEMA4D_LTR12C     ----------------------CTACCAATCAGCAGGAT-----GTGGGTGGGGCCAGATAAGAGAATAAAAGCAGGCTGCCCGAGCCAG 1088
TNFRSF10B_LTR12   --------------------CTCTACCAATCAGCAGGGT-----GTGGGTGGGGCCAGATAAGAGAATAAAAGCAGGCTGCCAGAGCCAG 1017
TP63_LTR12C       --------------------CTCTACCAATCAGCAGGAA-----GTGGGTGGGGCCAGATAAGAGAATAAAAGCAGGCTGCCCCAGCCAG 798
DHRS2_LTR12D_1    CATAAAA--------------TGGACCAATCAGCAGGAT-----GTGGGTGTGGCCAGTTAAGGGAATAAAAGCAGGCTGCCTGAGCCGG 732
DHRS2_LTR12D_2    ---------------------TGGACCAATCAGCAGGAT-----GTGGACAGGGCCAGATAAGGGAATAAAAGCAGGCCACCCGAGCCAG 241
NR1H4_LTR12C      --------------------CTCTACCAATCAGCAGGAT-----GTGGGTGGGGCCAGAAAAGAGAATAAAAGCAGGCTGCCCGAGCCAG 1005
C9orf53_LTR12CD   --------------------CTCTACCAATCAGCAGGAT-----GTGGGTGGGGCCAGATAAGAGAATAAAAGCAGGCTGCCGGAGGCAG 791
CPED1_LTR12C      -------------------GCTCTACCAATCAGCAGGAT-----GTGGGTGGGGCCAGATAAGAGAATAAAAGCAGGCTGTCCCAGCCAG 983
C9orf85_LTR12C    --------------------CTCTACCAATCAGCAGGAT-----GTGGGTGGGGCCAGAGAAGAGAATAAAAGCAGGCTGCCCCAGCCAG 964
CCR4_LTR12C       TGACAAAACAGGCCACTGGGCTCTACCAATCAGCAGGAT-----GTGGGTGGGGCCAGATAAGAGAATAAAAGCAGGCTGCCGGA-CCAG 1243
ACSBG1_LTR12C     --------------------CTCTACCAATCAGCAGGAT-----GTGGGTGGGGCCAGATAAGAGAATAAAAGCAGGCTGCCCGAGCTAG 1010
KCNN3_LTR12C      --------------------CTCTACCAATCAGCAGGAT-----GTGGGTGGGGCCAGATAAGAGAATAAAAGCAGGCTGCCTGAGCCAG 1037
CSF3_LTR12CD      --------------------CTCTACCAATCAGCAGGAT-----GTGGGTGGGGCCAGATAAGAGAATAAAAGCAGGCTGCCCGAGCCAG 1585
TMOD1_LTR12EB     ACCAGTCGGCTCTCTGTAAAATGGACCAATCAGCAGGAT-----ATGGGTGGGGCCAGATAAGAGAATAAAAGCAGGCTGCCGGATAACG 998
SLC36A2_LTR12     ---------------------TGGACCAATCAGCAGGAC-----GTGGGTAGGGCCAAATAAGGGAATAAAAGCTGGCCACTAGAGCCAG 417
IER3_LTR12C       ----------------------CTACCAATCAGCAGGAT-----GTGGGTGGGGCCAGATAAGAGAATAAAAGCAGGCTGCCCGAACCAG 969
PIK3C2G_LTR12C    --------------------CTCTACCAATCAGCAGGAT-----GTGGGTGGAGCCAGATAAAAGAATAAAAGCAGGCTGCCCAAGCCAG 793
CT49_LTR12E       TGTAAAA--------------TGGGCCAATCAGCAGGAT-----GTGGGTGGAGTCAGATAAGGGAATAAAAGCAGGCTGCCAGCTCCAG 1005
PTPN13_LTR12EC    --------------------CTCTACCAATCAGCAGGAT-----GTGGGTGGAGCCAGACAAGAGAATAAAAGCAGGCTGCCTGATCCAG 882
RADIL_LTR12C      --------------------CTCTACCAGTCAGCAGGAT-----GTGGGTGGGGCCAGAT--GAGAATAAAAGCAGGCTGCCCGAGCCAG 967
Clustal Consens                            **  ********        ***     * **       ***                      * 28

ADH1C_LTR12       CAGCAGCAACCCACTCAGGTCCCCTTCCATGCCATGGAAGCTTTCTTCTTTTGCTCTTC-GCAATAAATCTTG-----CTGCTGCCCACT 476
GBP5_LTR12C       CAGTGGCAACCCACTGAGGTCACCCTCCAGGCTGTGGAACCTTTGTTCTTTCACTCTTT-GCAATAAATCTTG-----CTGCTGCTCACT 1102
SEMA4D_LTR12C     CATTGGCAACCCGCTCGGGTCCCCTTCCACACTGTGGAAGCTTTGTTCTTTCGCTCTT--------------G-----CTACTGCTAGCT 1159
TNFRSF10B_LTR12   CAGTGGTAAC--------------------ACTGTGGAAGCTTCCTTCTTT--CCCTCT-GCAATAAATCTTG-----CTACTGCTCACT 1079
TP63_LTR12C       CAGTGGCAACCCGCTGGGGTCACCTTCCACACTGTGGAAGCTTTGTTCTTTTGCTCTTT-GCAGTAAATCTTG-----CTACTGCTCACT 882
DHRS2_LTR12D_1    CAGCAGCAACCTGCTCTGGTTCCCTTCCACGCTGTGGAAGCTTTGTTCTTTTGGTCTTC-ATGATAAATCTTG-----CTGCTGCTCACT 816
DHRS2_LTR12D_2    CAGCAGCAACCTGCTTGAGTCCCCTTCCACACTGTGGAAGTTTTGTTCTTTCGCTCTTT-GCAATAAATCTTGATTCACTGCTGCTCACT 330
NR1H4_LTR12C      CAGGGGCAACCCGCTCGGGTCACCTTCCACACTGTGGGAGCTTTGTTCTTTCGCTCTTT-GCAATAGCTCTTG-----CTACTGCTCACT 1089
C9orf53_LTR12CD   CATTGGCAACCCACTCGGGTCCCCTTCTGCACCGTGGAAGTTTTGTTCTTTCACTCTTT-GCAATAAATCTTG-----CTACTGCTCACT 875
CPED1_LTR12C      CAGTGGCAACCCGCTCCGGTCCCTTTTCACGTTGTGGAAGCTTTGTTCTTTTGCGCTTT-GCAATAAATCTTG-----CTGCTGCTCACT 1067
C9orf85_LTR12C    CAGTGGCAACCTGCTGGGGTCCCCTTCCACACTGTGGAAGCTTGGTTCTTT---------GCAATAAATCTTG-----CTGCTGCTCACT 1040
CCR4_LTR12C       CATTGGCAACCCGCTGGGGTCCCTTTCCACAGTGTGGAAGCTTTGTTCTTTTGCTCTTT-ACAACAAATCCTG-----CTACTGTTCGCT 1327
ACSBG1_LTR12C     CAGTGGCAACCCGCTCCGGTCACCTTCCACGCTGTGAAAGCTTTATTCTTTTGCTCTTT-GCAATAAATCTTG-----CTACTGCTCAGT 1094
KCNN3_LTR12C      CAGTGGCAACCTGCTTGGGTGCCCTTCCACACTGTGGAAGCTTTGTTCTTTTGCTCTTT-GCAATAAATCTTG-----CTACCGCTCACT 1121
CSF3_LTR12CD      CAGTGGCAACGGGCACAGGTCCCCATCCACAATATGGCAGCTTTGTTCTTTTGCTGTTT-GCGATAAATCTTG-----CTACTGCTCGCT 1669
TMOD1_LTR12EB     CAGTGGCAACCCGCTCGGGTCCACTTCCGCACTGTGGAAACTTTGTTCTTTCTCTGTTT-GCCATAAATCTTG-----CCACTGCTCACT 1082
SLC36A2_LTR12     CAGCAGCAACCCATTCGGGTCCCCTTCCAAGCTGTGGAAGCTTTGTTCTTTTGCTCTTC-ACAATAAATCTTG-----CTGCTGCTCACT 501
IER3_LTR12C       CATTGGCAACCCGCTTGGGTCCCTTTCCAGACTGTGGAAGGTTTGTTCTTTTGCTCTTT-GCAATAAGTCTTG-----CTGCTGTTCACT 1053
PIK3C2G_LTR12C    CAGTGGCAACCCACTCAGGTCCCCTTCCACACTGTGGAAGCTTTGTTCTCTCGCTCTTT-GCAATAAATCCTG-----CTGCTGCTCACT 877
CT49_LTR12E       CAGCGGCAACCTGCTCGGGTCCTCTTGTACTGTGTAGAAACCTTGTTCTTTCGGTCTTTTGCAATAAATCTTG-----CGGCTGCTCACT 1090
PTPN13_LTR12EC    CAGTGGCAACCCGCTCAGGTCCCCTTCCACACTGTGGAATCTTGGTTCTTTCACTCTTT-GCAATAAATCTTG-----CTGCTGCTCACT 966
RADIL_LTR12C      CAGTGACAACCCACTCGGGTCCCCTTCCACACCG-GGATGCTTTGTTTGTTCGCTCTTT-GCAATAAATCTTG-----CTGCTGCTCACT 1050
Clustal Consens   **     ***                                *  **   *                     *     *  * *     * 42

ADH1C_LTR12       CTTT-GGGTCCATGCTACCTTTATGAGCTGTAACACTCATGGCAAAGGTCTGCAGCTTCACTCCTGAAGCC------------------- 546
GBP5_LTR12C       CTTT-GGGTCCACACTGCCTTTATGAGCTGTAACACTCACTGGGAATGTCTGCAGCTTCACTCCTGAAGCC------------------- 1172
SEMA4D_LTR12C     CTTT-GGGTCCACGCTGCTTTTATGAGCTGTAACACTCACCGCGAAGATCTGCAGCTTCACTCCTGAGCCCAGCGAGACCACGAGCCCAC 1248
TNFRSF10B_LTR12   CTTT-GGGTCCACACTGCCTTTATGAGCTGTAACACTCACCTTGAAGGTCTGCAGCTTCACTCTTGAAGCC------------------- 1149
TP63_LTR12C       CTTT-GGGTGCACACTGCTTTTATGAGCTGTAACACTCACCGTGAAGGTCTGCAGCTTCACTCCTGAAGCC------------------- 952
DHRS2_LTR12D_1    CGTT-GGGTCCGTGCCACCTTTAAGAGCTGTAACACTCACCGCGAAGGTCTGCAACTTCACTCCTGGGGCC------------------- 886
DHRS2_LTR12D_2    CTTT-GGGTCCGCGCTGCCTTTAAGAGCTGTAACACTCACCACGAAGGTCTGCAACTTCACTCCTGAGGCC------------------- 400
NR1H4_LTR12C      CTTT-GGGTCCACACTGCTTTTATGAGCTGTAACGCTCACCGTGAAGGTCTGCAGCTTCACTCCTGAAGCC------------------- 1159
C9orf53_LTR12CD   CTTT-GGGTCCACGCCGCTTTTATGAGCTGTAACACTCAGTGCGAAGATCTGCAGCTTCACTCCTGAGCCC------------------- 945
CPED1_LTR12C      CTTT-GGGTCCACACTGCCTTTATGAGCTGTAACACTCACTGCGAAGATCTGCAGCTTCACTCCTGAAGCC------------------- 1137
C9orf85_LTR12C    CTTT-GGGTCTACACTGCCTTTATAAGCTTTAACACTCACCGCAAAGGTCTACAGCTTCATTCCTGAAGCC------------------- 1110
CCR4_LTR12C       CTTT-GGGTT--AACTGCTTTTATGAGCTGTAACACTCACTGTGACAATTTGCAGCTTCACTCCTCAGCCC------------------- 1395
ACSBG1_LTR12C     CTTT-GGGTCCACGCTGCTTTTATGAGCTGTAACACTCACTG-GAAAGTCTGCAGCTTCATTCCTGAAGCC------------------- 1163
KCNN3_LTR12C      CTTT-GGGTCCACACTGCTTTTATGAGCTGTAACACTCACTGTGAAGGTCTGCAGCTTTACTCCTGAAGCC------------------- 1191
CSF3_LTR12CD      TTTT-GGGTCCACACTGCTTTTATGAGCTGTAACACTCACCACGAAGGTCTGCAGCTTCACTCCTGAAGCC------------------- 1739
TMOD1_LTR12EB     CTTT-GGGTCCACACTGCCTTTATGAGTTGTAACACTCACCGCGAAGGTCTGCAGCTTCACTCCTGAAGCC------------------- 1152
SLC36A2_LTR12     CTTT-GGGGCCATACTACCTGTATGAGCTGTAACATTCGCTGCAAAGGTCTGCAGCTTCACTCCTGAAGTC------------------- 571
IER3_LTR12C       TTTTTGGGTCCATGCTGCTTTTATAAGTTGTAACACTTACTGCGAAGATCTGCAGCTTCACTCCTGAGCC-------------------- 1123
PIK3C2G_LTR12C    CTTT-GGGTCCACACTGCCTTTATGAGCTGCAACCCTCACCGCGAAGGTCTGTAGCTTCACTCCTGAAACC------------------- 947
CT49_LTR12E       CTTT-GGGTCCGCACTGCGTTTGTGAGCTGTAACATTTACCGCGAAGGTCTGCAGCTTCACTCCTGAAGCC------------------- 1160
PTPN13_LTR12EC    GCTT-GGGTCCACACTGCTTTTATGAGCTGTAACACTCACCGCGAAGGTCTGCAGCTTCACTCCTGAAGCC------------------- 1036
RADIL_LTR12C      CTTT-GGGTCCACACTGCCTTTATGAGCTATAACACTCATCGCAAAGATCTGCAGCTTCACTCCTAAAGCC------------------- 1120
Clustal Consens     ** ***      *  * * *   ** *  ***  *       *   * *  * *** * ** *                          69
```

```
ADH1C_LTR12        ----------------------------------------------------------------------------------AGCAAGGC 554
GBP5_LTR12C        ----------------------------------------------------------------------------------AGCGAGAC 1180
SEMA4D_LTR12C      CGGGAGGAACGAACAACTCCAGACGCGCTGCCTTAAGAGCTGTAACACTCACCGTGAAGGTCTGCAGCTTCACTCCCGAGCCAGCGAGAC 1338
TNFRSF10B_LTR12    ----------------------------------------------------------------------------------AGCGAGAC 1157
TP63_LTR12C        ----------------------------------------------------------------------------------AGCGAGAC 960
DHRS2_LTR12D_1     ----------------------------------------------------------------------------------AGCAAGAC 894
DHRS2_LTR12D_2     ----------------------------------------------------------------------------------AGTGAGAC 408
NR1H4_LTR12C       ----------------------------------------------------------------------------------AGCGAGAC 1167
C9orf53_LTR12CD    ----------------------------------------------------------------------------------AGCGAGAC 953
CPED1_LTR12C       ----------------------------------------------------------------------------------AGCGAGAC 1145
C9orf85_LTR12C     ----------------------------------------------------------------------------------AGCGAGAC 1118
CCR4_LTR12C        ----------------------------------------------------------------------------------AGCGAGAC 1403
ACSBG1_LTR12C      ----------------------------------------------------------------------------------AGCAAGAC 1171
KCNN3_LTR12C       ----------------------------------------------------------------------------------AGCGAGAC 1199
CSF3_LTR12CD       ----------------------------------------------------------------------------------ACTAAGAC 1747
TMOD1_LTR12EB      ----------------------------------------------------------------------------------ATCGAGAC 1160
SLC36A2_LTR12      ----------------------------------------------------------------------------------AGCAAGAC 579
IER3_LTR12C        ----------------------------------------------------------------------------------AGCAAGAC 1131
PIK3C2G_LTR12C     ----------------------------------------------------------------------------------AGCGGGAC 955
CT49_LTR12E        ----------------------------------------------------------------------------------AGTGAGAT 1168
PTPN13_LTR12EC     ----------------------------------------------------------------------------------AGCGAGAC 1044
RADIL_LTR12C       ----------------------------------------------------------------------------------AGCAAGAC 1128
Clustal Consens                                                                                      *    *   71

ADH1C_LTR12        CATGAACCCACCAGG--AGGAACAAACAACTCTGGACGTGCCATG--------------------------------------------- 597
GBP5_LTR12C        CACGAACCCACCAGG--AGGAACAAACAACTCCAGACGCGCAGCC--------------------------------------------- 1223
SEMA4D_LTR12C      CACGAACCCACCAGA--AGGAAGAAACT-------------------------------------------------------------- 1364
TNFRSF10B_LTR12    CACGAGCCCACCAGG--AGGAAAGAACAACTCCAGACCCACTGC---------------------------------------------- 1199
TP63_LTR12C        CAGGAGTCCACTGGG--AGGAACGAACAACTCCAGACGCACCGC---------------------------------------------- 1002
DHRS2_LTR12D_1     CACGAATGCACCGAG--AGGAATGAAC--------------------------------------------------------------- 919
DHRS2_LTR12D_2     CACAAACCCTCCAGA--AGGAATGAACAACTCCAGACGCACTGCC--------------------------------------------- 451
NR1H4_LTR12C       CACGAGCCCACCGGG--AGGAAGGAACAACTCCAGACGCGCTACA--------------------------------------------- 1210
C9orf53_LTR12CD    CACGAGCCCATGGAG--AGGAACAAACAACTCCAGACGCGCTGCGTTAAGAGCTGTAACATTCACCGCGAAGGTCTGCAGCTTCACTCCT 1041
CPED1_LTR12C       CACGAGCCCCCCGGG--AGGAACGAACAACTCCAGACGCGCCAC---------------------------------------------- 1187
C9orf85_LTR12C     CACAAGCCCACCGGG--AGCAATAAACAACTCCAGACGCGCCGC---------------------------------------------- 1160
CCR4_LTR12C        CACGAGCCCACTGAG--AGGAACGAACAACTCCAGACGCACTGGT--------------------------------------------- 1446
ACSBG1_LTR12C      CGCGAGCCCCCCGGGGGAGGAATGAACAACTCCAGAGACGCCAC---------------------------------------------- 1215
KCNN3_LTR12C       CACGAGCCCACTGGG--AGGAACGAACAATTCCAGACGCACTGC---------------------------------------------- 1241
CSF3_LTR12CD       CACGAGCCCACCGGG--AGGAATGAACAACTCCGGCCGCGCTGC---------------------------------------------- 1789
TMOD1_LTR12EB      CACGAACCCACTGGG--AGGAACAAACAACTCCAGAGGCTCCGC---------------------------------------------- 1202
SLC36A2_LTR12      CATGAACCCACTGGG--AGGAACAAACAACTCTGGACGCGCCACC--------------------------------------------- 622
IER3_LTR12C        CATGAACCTACCAGA--AGGAAGAAACT-------------------------------------------------------------- 1157
PIK3C2G_LTR12C     CACGAACCCACCGGG--AGGAACGAACAACTCCAGACACACCGC---------------------------------------------- 997
CT49_LTR12E        CACGGACCCACCAGA--AGGAAGAAACT-------------------------------------------------------------- 1194
PTPN13_LTR12EC     CACGAACCCACCGGG--AGGAACAAACAACTCCAGACGCGCTGC---------------------------------------------- 1086
RADIL_LTR12C       CACGAACCCACTGGG--AGGAACGAACAACTCCAGACGCGCCGC---------------------------------------------- 1170
Clustal Consens    *                ** **  ***                                                                79

ADH1C_LTR12        -------------------------------------------------------------------------------------TTTAA 602
GBP5_LTR12C        -------------------------------------------------------------------------------------TT-AA 1227
SEMA4D_LTR12C      ------------------------------------------------------------------------------------------ 1364
TNFRSF10B_LTR12    -------------------------------------------------------------------------------------CTTAA 1204
TP63_LTR12C        -------------------------------------------------------------------------------------CTTAA 1007
DHRS2_LTR12D_1     ------------------------------------------------------------------------------------------ 919
DHRS2_LTR12D_2     -------------------------------------------------------------------------------------TTTAA 456
NR1H4_LTR12C       --------------------------------------------------------------------------------------TTAG 1214
C9orf53_LTR12CD    GAGCCAGCGAGACCAGGAACCCACCAGAAAGAAGAAGCTCCGAACACATCTGAACATCAGAAGGGAGAGACTCCAGACGCGTCATCTTAA 1131
CPED1_LTR12C       -------------------------------------------------------------------------------------CTTAA 1192
C9orf85_LTR12C     -------------------------------------------------------------------------------------CTTAA 1165
CCR4_LTR12C        -------------------------------------------------------------------------------------TTAAG 1451
ACSBG1_LTR12C      -------------------------------------------------------------------------------------TTTAA 1220
KCNN3_LTR12C       -------------------------------------------------------------------------------------CTTAA 1246
CSF3_LTR12CD       -------------------------------------------------------------------------------------CTTAA 1794
TMOD1_LTR12EB      -------------------------------------------------------------------------------------CTTAA 1207
SLC36A2_LTR12      -------------------------------------------------------------------------------------TTTAA 627
IER3_LTR12C        ------------------------------------------------------------------------------------------ 1157
PIK3C2G_LTR12C     -------------------------------------------------------------------------------------CTTAA 1002
CT49_LTR12E        ------------------------------------------------------------------------------------------ 1194
PTPN13_LTR12EC     -------------------------------------------------------------------------------------CTTAA 1091
RADIL_LTR12C       -------------------------------------------------------------------------------------CTTAA 1175
Clustal Consens                                                                                               79

ADH1C_LTR12        GAGCTGTAACACTCACTGTGAAGGTCTGCAGCTTCACTCCTGAAGTCAGTGAGACCATGAAGCC-ACTGGGAGGAATGAAC--------- 682
GBP5_LTR12C        GAGCTGTAACACTCACCGCGAAGGTCTGCAGCTTCACTCCTGA-GCCAGCCAGACCACGAACCC-ACCAGAAGGAAGAAACTCC------ 1309
SEMA4D_LTR12C      ----------------------------------------------------------------------------------CT------ 1366
TNFRSF10B_LTR12    GAGCTGTAACACTCACTGGGAAGGTCTGCAGCTTCACTCCTGA-GCCAGTGAGACCACGAACCC-ACCAGAAGGAAGAAACTCC------ 1286
TP63_LTR12C        GAACTTCAACACTCACTGCGAAGGTCTGCAGCTTCACTCCTGA-GCCAGCGAGACCACGAACCC-ACCGTAAGGAAGAAACTCC------ 1089
DHRS2_LTR12D_1     ------------------------------------------------------------------------------------------ 919
DHRS2_LTR12D_2     GAGCTGTAACACTCACTGCGAAGGTCTGCAGCTTCACTCCTGAAGCCAGCAAGACCACTAACCC-ACCAGGAGGAATGAACAACTCCGGA 545
NR1H4_LTR12C       GAGCTCTAACACTAACAGCGAAAGTCTGCAGCTTCACTCCTGA-GCTAGCAAGACCACGAACCC-ACCAGAAGGAAGAAACGCC------ 1296
C9orf53_LTR12CD    GAGCTGTAACACTCACGGCGAAGGTCTGCAGCTTCACTCCTGA-GCCAACGAGACCACGAACCC-ACCAGAAGGAAGAAACTCC------ 1213
CPED1_LTR12C       GAGCTGTAACACTCACCGCGAAGGTCTGCAGCTTCACTCCTGA-GCCAGCGAGACCACGAACCC-ACCAGAAGGAAGAAACTCC------ 1274
C9orf85_LTR12C     GAGCTGTAACGCTCACCGTGAAGGTCTGCAGCCTCACTCTTGA-GCCAGCGAGACCACGAACCC-ACCAGAAGGAAGAAACTCC------ 1247
CCR4_LTR12C        AGCTGTAACACTTAACGAAGAAAGTCTGCAGCTTCACTCCTGA-GCCAGCGAGACCCCGACCAC-ATCT-------GAGACCCT------ 1526
ACSBG1_LTR12C      GAGCTGTAACACTCACCGTGAAGGTCTGCAGCTTCACTCCTGA-GCCAGCGAGACCACGAACCC-ACCAGAAGGAAGAAACTCC------ 1302
KCNN3_LTR12C       GAGCTGTAACACTCACCGCAAAGGTCTGCAGCTTCACTCCTGA-GCCAGGGAGTCCACGAACCC-ACCAGAAGGAAGAAACTCC------ 1328
CSF3_LTR12CD       GAGCTATAACACTCACCGCGAAGGTCTGCAGCTTCACTCCTCA-GCCAGCGAGACCACGAACCC-ACCAGAAGGAAGAAACTGC------ 1876
TMOD1_LTR12EB      GAGCTGTAACACTCACCGCGAAGGCCTGCAGCTTCACTCCTGA-TCCAG-GAGACCACAAACCC-ACCGGAAGGAAGAAACTCC------ 1288
SLC36A2_LTR12      GAGCTGTAACACTCACTGCAAAGGTCTGCAGCTTCACTCCTGAAGTCAGCAAGACCACGAACCC-ACCAGAAGGAAGAAACTCC------ 710
IER3_LTR12C        ----------------------------------------------------------------------------------CC------ 1159
PIK3C2G_LTR12C     GAGCTGTAACACTCACCGCGTAGGTCCGCAGCTTCACTCCTGA-GCCAGCGAGACCACGAACCC-ACCAGAAGGAAGAAACTCC------ 1084
CT49_LTR12E        ----------------------------------------------------------------------------------CC------ 1196
PTPN13_LTR12EC     GAGCTGTAACACTCACCACAAAGGTCCGCAGTTTCACTCCTGA-GCCAGCGAGACCGCGAACCCCACCAGAAGGAAGAAACTCC------ 1174
RADIL_LTR12C       GAGCTGTAACACTCACCGCGAAGGTCTGCAGCTTCACTCCTGA-GCCAGCGAGACCACGAACCC-ACCAAAAGGAAGAAACTCC------ 1257
Clustal Consens                                                                                               79
```

```
ADH1C_LTR12      -------------------------------------AACTCTGGACATGTCACCTTTAAGAGCTCTGACACTCACTGCGAAGGTCTGCA 735
GBP5_LTR12C      ----------AAACACATCCGAACATCAGAAGGAGCAAACTCCTGACACGCCACCTTTAAGAACCGTGACACTCAACGCTAGGGTCCGCG 1389
SEMA4D_LTR12C    ----------GAACACATCTGAACATCAGAAGGGACAGACTCCAGACGCGCCACCGT-AAGAGCTGTAACACTCACCGCGAGGGTCCACG 1445
TNFRSF10B_LTR12  ----------GAACACATCCGAACATCAGAAGGAACAAACTCCAGACACGCCGCCTTTAAGAACTGTAACACTCACCGCGAGGGTCCGAG 1366
TP63_LTR12C      ----------GAACACATCCGAACATCAGAAGGAACAAACTCCAGACGCGCCACCTT-AAGAGCTGTAACACTCACCGCCAGGGTCCGCG 1168
DHRS2_LTR12D_1   -------------------------------------AACTCTGGACACACCATCTTTAAGAACCGTAATACTCACCGCAAGGGTCTGCA 972
DHRS2_LTR12D_2   CGGGAGGAATGAACAACTCCGGATGGGAGGAATGAACAACTCCGGACACACCATCTTTAAGAACTGTAACACTCATTGCGAGGGTCCGTG 635
NR1H4_LTR12C     ----------GAACACATCCGAACATCGGAAGGAACAAACTCCAGACGCGCCACCTT-AAGAGCTGTAACACTCACCGCGAGGGTCCGCG 1375
C9orf53_LTR12CD  ----------GAACACATCTGAACATCAGAAGGGACAGACTCCAGACGCACCACCTT-AAGAGCTGTAACACTCACCGCGAGGGTCCGCG 1292
CPED1_LTR12C     ----------GAACACATCTGAATATCAGAAGGGGCAAACTCCGGACACGCCGCCTTTAAGAACTGTAACACTCACTGTGAGGGTCCGCG 1354
C9orf85_LTR12C   ----------GAGCACATCTGAACATCAGAAGGAACAAACTCCAGACACGCCGCCTTTAAGAACTGTAACACTCACCGCGAGGGTCCATG 1327
CCR4_LTR12C      ----------GAGCACATCTGAACATAAGAAAGGACAAACTCCAGGCGCGCCACCTT--AAGAGTTTAACAGTCACCGCGAGGGTCCGAG 1604
ACSBG1_LTR12C    ----------GAACACATCCGAACGTCAGAAGGAACAAACTCCAGACGCGCCCCCTT-AAGAGCTGTAACACTCACCGCGAGGGTCTGCG 1381
KCNN3_LTR12C     ----------AAACACATCCGAACATCAGAAGGAACAAACTCCAGACGTGCCACCTT-AAGGGCTGTAACACTCACCGTGAGGGTCCGCG 1407
CSF3_LTR12CD     ----------GAACACATCTGAACATCAGAAGGAACAAACTCCAGATGCACCACCTT-AAGAGCTGTAACACTCACTGCGAGGGTCCGCG 1955
TMOD1_LTR12EB    ----------TAACACATCCGAACATCAGAAGGAACAAACTCCGGACACACCGCCTTTAAGAACTATAACACTCACCTCGAGGGTCCGCG 1368
SLC36A2_LTR12    ----------GGACACATCTGAACATCTGAAGGAACAAACTCCGGACACACCATCTTTAAGAACTGTAACACTCACCGCGAGGGCCCGTG 790
IER3_LTR12C      ----------GAACACATCTGAACATCAGAAAGGACAGACTCCAGAAGCGCCACCTT-AAGGGCTATAACACTCACCGCGAGGGTCTGTG 1238
PIK3C2G_LTR12C   ----------GAACACATCCGAACATCAGAAAGAACAAACTCCAGACGCGCCACCTT-AAGAGCTGTAACACTCACCGCGAAGGTCCGCG 1163
CT49_LTR12E      ----------AAACGCGGCCGAACATCAGAAGGAACAAACTCTGGACACACCATCTTTAAGAACTGTAACACTCACCGCACGTGTCCGCG 1276
PTPN13_LTR12EC   ----------AAACACATCCGAACATCAGAACGAACAAACTCCGGACATGCCACCTTTAAGAACTGTAACACTCACCACGAGGGTCCACA 1254
RADIL_LTR12C     ----------GAACACATCCGAACATCATAAAGAACAAACTGCGGACACGCAGCCTTTAAGAACTGTAACACTCACCGCGAGGGTCCGCG 1337
Clustal Consens                                        ***   *         * *  *      * * * ***        * *      94

ADH1C_LTR12      GCTT----------------------------------------------------------- 739
GBP5_LTR12C      GCTTCATTCTTGAAGTCAGTGAGACCAAGAAC----------CCACCAATTCCGGACA----- 1437
SEMA4D_LTR12C    GCTTCATTCTTGAAGTCAGTGAGACCAAGAAC----------CCACCAATTCCGGACAC---- 1494
TNFRSF10B_LTR12  GCTTCATTCTTGAAGGCAGTGAGACCAAGAAC----------CCACCAATTCCGGACAC---- 1415
TP63_LTR12C      GCTTCATTCTTGAAGTCAGAGAGACCAAGAAC----------CCACCAATTCCGGACAC---- 1217
DHRS2_LTR12D_1   ACTTCATTCTTGAAGTCAGTGAGGCCAAGAACCCA----------TCAATTCCGTACACATTT 1025
DHRS2_LTR12D_2   GCTTCATTCTTGAAGTCAGCGAGACCAAGAAC----------CCACCAATTCCAGACACA--- 685
NR1H4_LTR12C     GCTTCATTCTTGAAGTCAGTGAGACCAAGAAC----------CCACCAATTCCGGACACA--- 1425
C9orf53_LTR12CD  GCTTCATTCTTGAAGTCAGTGAGACCAAGAAC----------CCACCAATTCCGGACACA--- 1342
CPED1_LTR12C     GCTTCATTCTTGAAGTCAG----ACCAAGAAC----------CCACCAATTCCAGACACA--- 1400
C9orf85_LTR12C   GCTTCATTCTTGAAGTCAGTGAGACCAAGAAC----------CCACCAATTCCGGATACA--- 1377
CCR4_LTR12C      ATTTCATTCTTGAAGTCGGTGAGACCAAGAACCCA----------CTAATTCCAGATACA--- 1654
ACSBG1_LTR12C    GCGTCAGTCTTGAAGTCAGTGAGACCAAGAAC----------CCACCAATTCGGGACAC---- 1430
KCNN3_LTR12C     GCTTCATTCTTGAAGTCAGTGAGACCAAGAAC----------CCACCAATTCCGGACACA--- 1457
CSF3_LTR12CD     GCTTCCTTCTTGAAGTCAGTGAGACCAAGCACTCA----------CCAGTTTCGGACACA--- 2005
TMOD1_LTR12EB    GCTTCATTCTTGCAGTCAGTGAGACCAAGAAC----------CCACCAATTCCGGACA----- 1416
SLC36A2_LTR12    GCTTCATTCTTGAAGTCAGCAAGACCAAGAACCCACCAGAAGGAACCAATTCCGGACAC---- 849
IER3_LTR12C      GCTTCGTTCTTGAAGTCAGTGAAACCAAGAACCCA----------CC---------------- 1275
PIK3C2G_LTR12C   GCTTCATTCTTGAAGTCAGTGAGACCAGGAACCCA----------GCAATTCCGGACGCA--- 1213
CT49_LTR12E      GCTTCATTCTTGAAGTCAGTGAGACCAAGAACCCA----------CCA--------------- 1314
PTPN13_LTR12EC   GCTTCATTCCTGAAGTCAGTGAGACCAAGAAC----------CCACCAATTCCGGACACA--- 1304
RADIL_LTR12C     ACTTCATTCTTGAGGTCAGTGAGACCAAGAAC----------CCACCAATTCCGGACACA--- 1387
Clustal Consens     *                                                            95
```

Corresponding to Figure 4A.

Figure S2: Predicted NF-Y binding sites within LTR12

Alignment of twenty-two LTR12 promoter sequences that regulate the expression of adjacent host genes and respond to treatment with HDAC inhibitors. Putative NF-Y binding sites were identified by in-silico prediction using the ALGGEN PROMO tool (http://alggen.lsi.upc.es/) [61, 62]). To visualize their position, these sites were marked green in the alignment, whereas TATA boxes (AATAAA) were highlighted in orange. If two predicted binding sites for NF-Y overlapped, one site was highlighted in a lighter green and underlined.

Corresponding to Figure 4A.

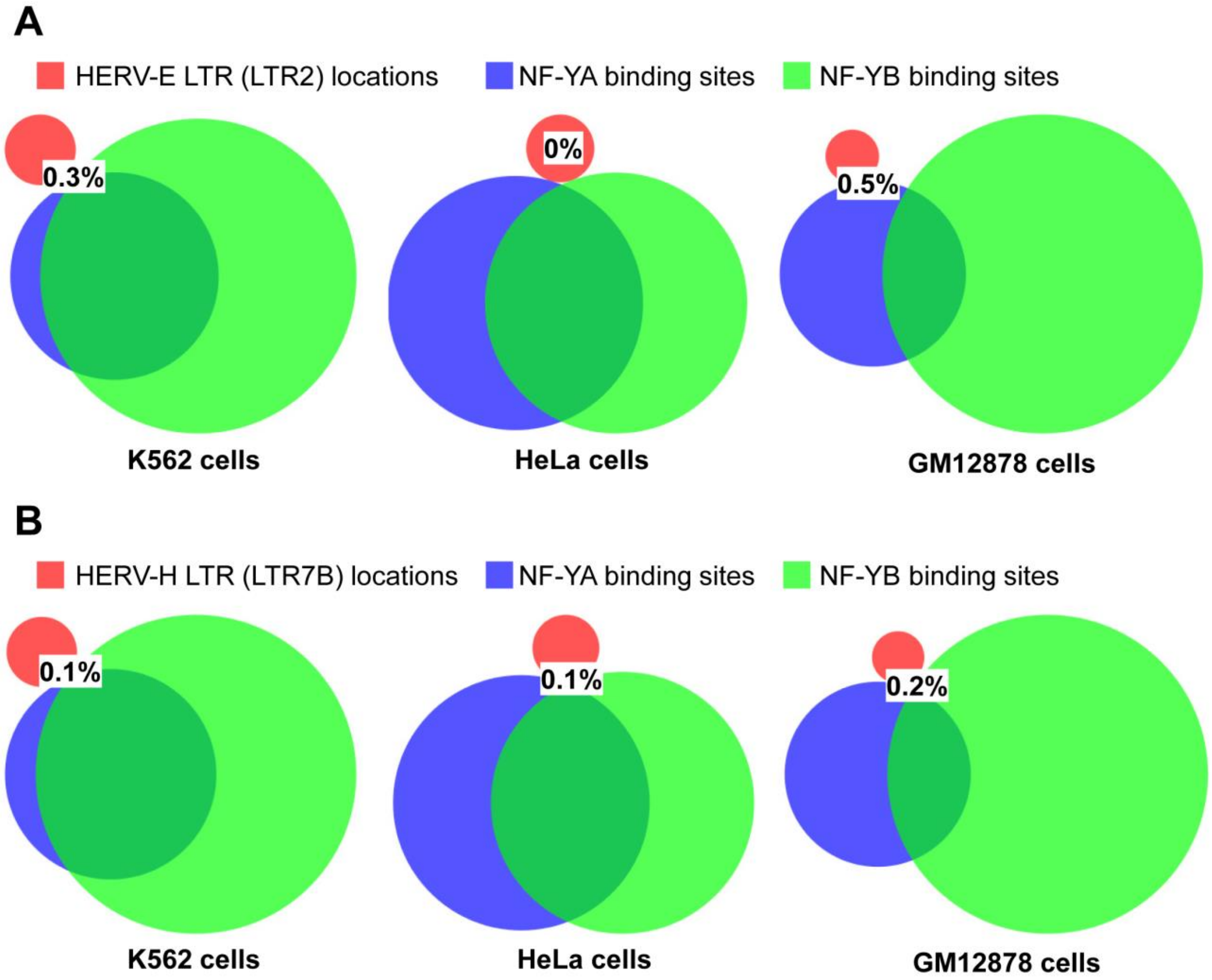

Corresponding to Figure 4B.

Figure S3: Low representation of consensus NF-Y binding sites within the LTRs of endogenous retroviruses other than ERV9

ChIP-seq data for binding of the NF-Y subunits alpha (NF-YA) and beta (NF-YB) according to [27] were retrieved from GEO and analyzed to identify LTRs from the HERV-E family, termed LTR2 according to Repbase [21], **[A]**, and from the HERV-H family, termed LTR7B, **[B]**, which

control the expression of cellular genes e.g. *APOC1*, *GSDMB* and *DNAJC15* (cf. Figure 2B). The results from K562 cells, HeLa-S3 cells and GM12878 cells are shown. The binding of NF-Y to the LTRs differs between the three cell lines. In total only 0% - 0.5% of the analyzed LTR2s and 0.1 - 0.2% of the analyzed LTR7Bs were bound by NF-Y in cells.

Corresponding to Figure 4B.

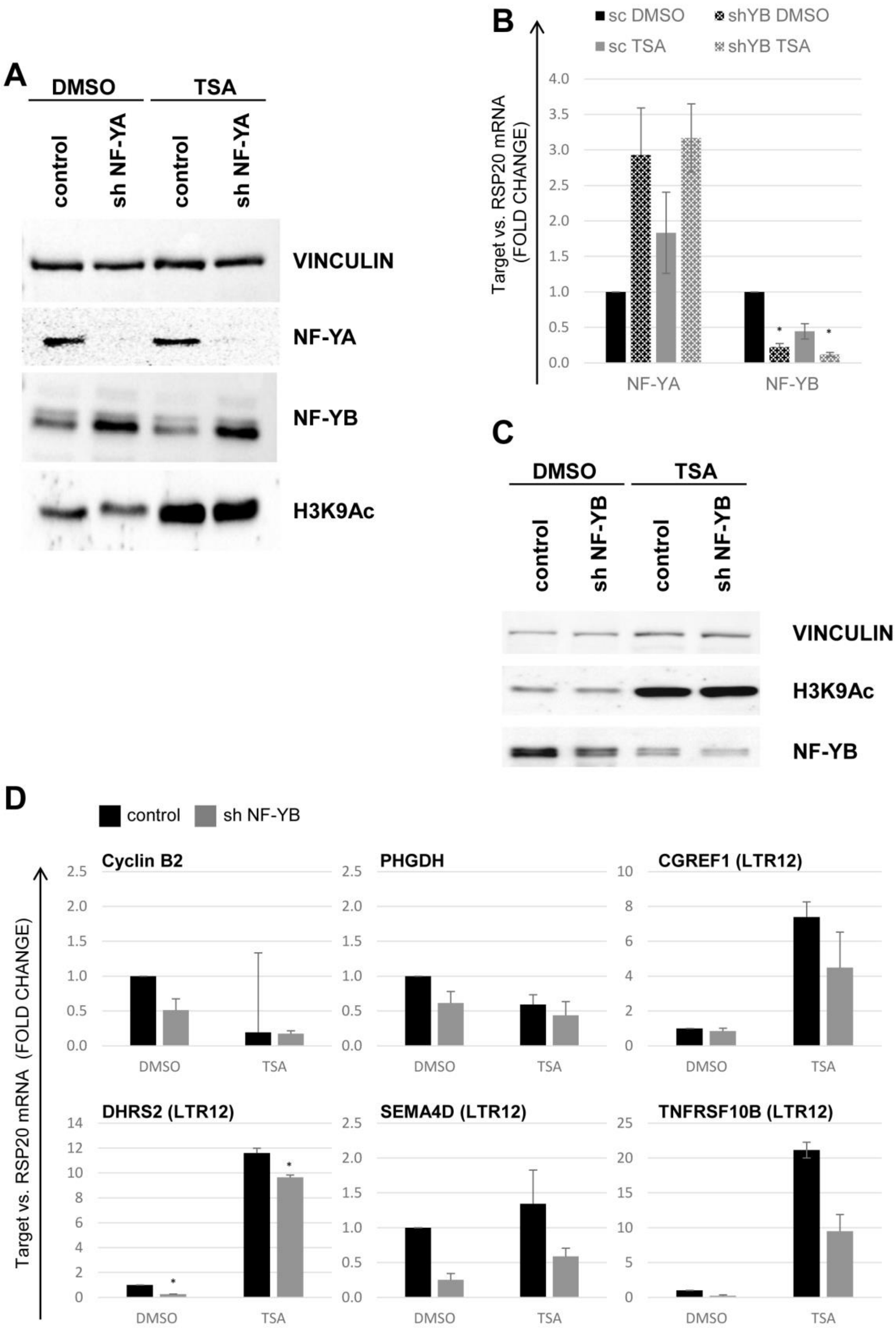

Corresponding to Figure 6.

Figure S4: NF-Y depletion by shRNA

HeLa cells were transduced to deplete NF-Y subunits by shRNA as described [49, 64]. **[A]** Cells were treated with 2 µM TSA or DMSO for 18 h. Immunoblot analysis confirmed that NF-YA was efficiently depleted. Note that NF-YB levels were increased in response to the depletion of NF-YA, perhaps reflecting an as yet uncharacterized cellular mechanism to regulate the levels of NF-Y subunits. **[B]** Total RNA was isolated and reverse transcribed into cDNA. Next, relative gene expression was assessed by qRT-PCR. The mRNA levels corresponding to NF-YB are depicted and were found significantly decreased by the corresponding shRNA. Note that NF-YB depletion increases the levels of NF-YA as found earlier [49]. mRNA levels were normalized to *RSP20*. Error bars represent SD (n=2). * = $p<0.05$, ** = $p<0.01$, *** = $p<0.001$. **[C]** as in [A] but using shRNA to deplete NF-YB. **[D]** The cells were transduced to deplete NF-YB and then treated with 2 µM TSA for 18 h, and relative gene expression was assessed by qRT-PCR with normalization to *RSP20*. The transcription levels of *Cyclin B2*, *PHGDH* as well as four LTR12-driven genes *(CGREF1, DHRS2, SEMA4D, TNFRSF10B)* are depicted. Removal of NF-YB resulted in an overall decreased transcription for LTR12-driven isoforms, which was significant for *DHRS2*, although the removal of NF-YA led to more pronounced downregulation of mRNA levels in most cases (cf. Figure 6). SD (n=2). * = $p<0.05$, ** = $p<0.01$, *** = $p<0.001$.

Corresponding to Figure 6.
